# AKR1C3-dependent lipid droplet formation confers hepatocellular carcinoma cell adaptability to targeted therapy

**DOI:** 10.7150/thno.74974

**Published:** 2022-11-07

**Authors:** Changqing Wu, Chaoliu Dai, Xinyu Li, Mingju Sun, Hongwei Chu, Qiuhui Xuan, Yalei Yin, Chengnan Fang, Fan Yang, Zhonghao Jiang, Qing Lv, Keqing He, Yiying Qu, Baofeng Zhao, Ke Cai, Shuijun Zhang, Ran Sun, Guowang Xu, Lihua Zhang, Siyu Sun, Yang Liu

**Affiliations:** 1 Department of Gastroenterology, Shengjing Hospital of China Medical University, Shenyang 110004, China.; 2 Innovative Research Center for Integrated Cancer Omics, Shengjing Hospital of China Medical University, Shenyang 110004, China.; 3 (CAS) Key Laboratory of Separation Science for Analytical Chemistry, Dalian Institute of Chemical Physics, Chinese Academy of Sciences, Dalian 116023, China.; 4 Department of Hepatobiliary and Splenic Surgery, Shengjing Hospital of China Medical University, Shenyang 110004, China.; 5 School of Life Science, Dalian University, Dalian 116023, China.; 6 Department of Hepatobiliary and Pancreatic Surgery, The First Affiliated Hospital of Zhengzhou University, Zhengzhou 450052, China.; 7 National Engineering Laboratory for Internet Medical System and Application, The First Affiliated Hospital of Zhengzhou University, Zhengzhou 450052, China.

**Keywords:** Lipid homeostasis, Lipid droplets, Lipophagy, Metabolic reprogramming, Mitochondrial dysfunction.

## Abstract

**Rationale:** Increased lipid droplet (LD) formation has been linked to tumor metastasis, stemness, and chemoresistance in various types of cancer. Here, we revealed that LD formation is critical for the adaptation to sorafenib in hepatocellular carcinoma (HCC) cells. We aim to investigate the LD function and its regulatory mechanisms in HCC.

**Methods:** The key proteins responsible for LD formation were screened by both metabolomics and proteomics in sorafenib-resistant HCC cells and further validated by immunoblotting and immunofluorescence staining. Biological function of AKR1C3 was evaluated by CRISPR/Cas9-based gene editing. Isotopic tracing analysis with deuterium^3^-labeled palmitate or carbon13-labeled glucose was conducted to investigate fatty acid (FA) and glucose carbon flux. Seahorse analysis was performed to assess the glycolytic flux and mitochondrial function. Selective AKR1C3 inhibitors were used to evaluate the effect of AKR1C3 inhibition on HCC tumor growth and induction of autophagy.

**Results:** We found that long-term sorafenib treatment impairs fatty acid oxidation (FAO), leading to LD accumulation in HCC cells. Using multi-omics analysis in cultured HCC cells, we identified that aldo-keto reductase AKR1C3 is responsible for LD accumulation in HCC. Genetic loss of AKR1C3 fully depletes LD contents, navigating FA flux to phospholipids, sphingolipids, and mitochondria. Furthermore, we found that AKR1C3-dependent LD accumulation is required for mitigating sorafenib-induced mitochondrial lipotoxicity and dysfunction. Pharmacologic inhibition of AKR1C3 activity instantly induces autophagy-dependent LD catabolism, resulting in mitochondrial fission and apoptosis in sorafenib-resistant HCC clones. Notably, manipulation of AKR1C3 expression is sufficient to drive the metabolic switch between FAO and glycolysis.

**Conclusions:** Our findings revealed that AKR1C3-dependent LD formation is critical for the adaptation to sorafenib in HCC through regulating lipid and energy homeostasis. AKR1C3-dependent LD accumulation protects HCC cells from sorafenib-induced mitochondrial lipotoxicity by regulating lipophagy. Targeting AKR1C3 might be a promising therapeutic strategy for HCC tumors.

## Introduction

Hepatocellular carcinoma (HCC) is the predominant form of primary liver cancer, constituting 75-85% of liver cancer cases 1, 2. Recent studies have shown that sorafenib, a commonly used first-line drug for advanced HCC, decreased oxidative phosphorylation (OXPHOS), implying that HCC cells require alternative energetic sources and metabolic pathways to support their survival in response to long-term sorafenib treatment 3, 4. Consistently, increased glycolytic flux critically contributes to sorafenib resistance, as inhibition of Hexokinase 2 (HK2) by 2-DG sensitizes resistant HCC cells to sorafenib 5, 6. These findings suggest the metabolic shift from OXPHOS towards glycolysis is critical for acquired sorafenib resistance. However, since HCC cells are highly reliant on fatty acid oxidation (FAO, β-oxidation) for energy production 7, 8, increased demand for lipid storage should be critically involved in facilitating HCC tumor adaptation to sorafenib therapy.

Lipid Droplets (LDs) are dynamic intracellular organelles that either store excess lipids or fuel cells with essential lipids to maintain lipid homeostasis 9. LDs are composed of a neutral lipid core, including triglycerides (TAGs or TGs) and sterol esters, surrounded by a phospholipid monolayer mainly composed of phosphatidylcholine (PC) and a broad range of proteins mainly involved in lipid metabolism 10. In eukaryotes, TAGs are synthesized predominantly in the ER. This biological process is mainly conducted by two enzymes, diacylglycerol acyltransferase 1(DGAT1) and diacylglycerol acyltransferase 2 (DGAT2) 11, 12. LDs serve as lipid reservoirs for energy production or membrane synthesis 13. Lipid storage via LDs is critical for protecting cells from lipotoxicity due to the buildup of excess lipids, such as fatty acids (FAs), toxic glycerolipids, and sterols, in cell membranes 14-16. In contrast, LD breakdown can be achieved by one of two lipolytic pathways, lipolysis or lipophagy. Lipolysis is mediated by adipose triglyceride lipase (ATGL) whereas lipophagy is the autophagic degradation of LDs 17. Fatty acids released from LD breakdown can be utilized either for mitochondrial β-oxidation, or biosynthesis of membrane phospholipids 9.

LD accumulation has been frequently observed in many types of cancer, emerging as a new hallmark of human malignancies 18, 19. In tumor cells, the intracellular excess lipid can be stored in LDs to prevent lipotoxicity and facilitate cell survival 11. Moreover, LD metabolism contributes to membrane phospholipid homeostasis owing to the requirement for the rapid proliferation of tumor cells 20. In addition, LDs can function as an energy source to support tumor invasion and migration via β-oxidation 21, 22. Recently, the role of LDs in mediating chemoresistance has been shown in some studies 18, 23, 24. However, the role of LDs in mediating drug resistance and its regulatory mechanisms are not understood in HCC tumors.

In this study, we aimed to identify the key modulator responsible for LD and energy homeostasis when exposed to drug treatment. We demonstrated that HCC cells inhibit FAO in response to long-term sorafenib, leading to LD accumulation. The intracellular excess FAs, which could not be degraded in mitochondria, are fueled into LDs via aldo-keto reductase family 1 member C3 (AKR1C3) activity. The AKR1C3-dependent LD metabolism effectively reduces cellular lipotoxicity and ROS generation, promoting HCC cell survival. Mechanistically, we show that AKR1C3 promotes LD formation by inhibiting autophagy-dependent LD degradation. Finally, AKR1C3 can directly control the metabolic switch between FAO and glycolysis, promoting the “Warburg effect” in HCC cells. Our findings not only reveal a critical role of AKR1C3 in lipid and energy homeostasis but provide a novel insight into the mechanism by which liver cancer cells respond to the increased demand of lipid storage.

## Results

### Metabolic shift from FAO to glycolysis leads to LD accumulation in sorafenib-resistant clones

**Figure 1:**
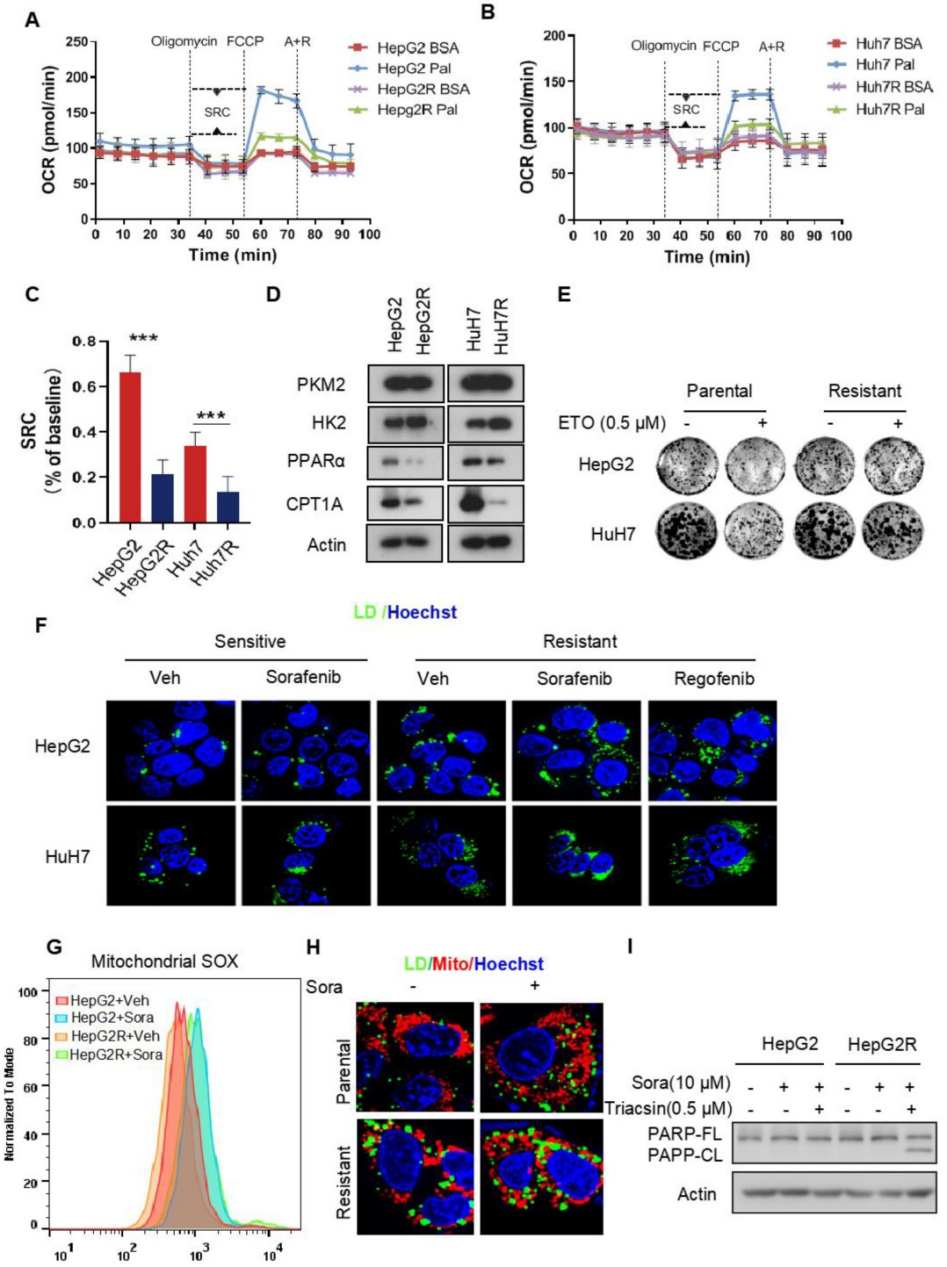
** Impairment of FAO induces LD accumulation in HCC cells (A-B)** FAO activity was measured under basal conditions by Seahorse XF-96 assays in the two paired HCC cell lines. Oligomycin, FCCP, antimycin, and rotenone were added at the indicated time points in the presence of either BSA or palmitate.** (C)** The percentage of maximum OCR after FCCP injection of baseline OCR (SRC) in these two paired cell lines. **(D)** Total cell lysates of HepG2, HepG2R, HuH7, and HuH7R lines were extracted and subjected to immunoblotting against the indicated antibodies. **(E)** The two paired HCC cell lines were treated with or without 25 μM Etomoxir for 2 weeks. Colony forming ability was determined by crystal violet assay. **(F)** HepG2 and HepG2R cells were treated with or without sorafenib or regorafenib (rego) for 24 h. Bodipy493/503 stained LDs were visualized and analyzed by immunofluorescence microscopy. Nucleus was stained by Hoechst (blue). **(G)** Flow cytometry histograms and the corresponding quantification of fluorescent intensity. **(H)** HepG2 and HepG2R cells were treated with or without 5 μM sorafenib for 24 h, respectively. Cells were stained by Bodipy493/503 and Mito-Tracker CMXROS and then subjected to microscopy. Representative images were shown: Cellular LDs (green); Mitochondria (red); and Nucleus (blue).** (I)** HepG2 and HepG2R cells were treated with 10 μM sorafenib and 0.5 μM Triacsin C for 12 h. The cell lysates were extracted and subjected to an immunoblotting assay.

Recent studies have shown that enhanced glycolysis contributes to acquired sorafenib resistance in HCC cells 25, 26. To determine the energetic metabolism in sorafenib-resistant HCC cells, we established two HCC cell lines with acquired sorafenib resistance, HepG2R and HuH7R, referring to the previously described approaches 27 (Figure S1A-B). The oxygen consumption rate (OCR) and extracellular acidification rate (ECAR) were measured to assess the mitochondrial respiration capacity and glycolysis, respectively, using a seahorse XF system. We found that the OCR dramatically decreased in both HepG2R and HuH7R cells, regardless of sorafenib status, compared to their parental HepG2 and HuH7 cells, respectively (Figure S1C-D). The impairment of OXPHOS was further evaluated by quantification of the spare respiratory capacity (SRC), showing that OCR decreased by ~7-fold in HepG2R cells and ~4-fold in HuH7R cells (Figure S1E). In contrast, the ECAR significantly increased in HepG2R and HuH7R cells compared to their parental lines (Figure S1F-G). Similarly, the ECAR/OCR ratio increased by ~3.4-fold and ~2.9-fold in HepG2R and HuH7 cell lines, respectively (Figure S1H). In addition, glycolytic flux was further examined using ^13^C_6_ glucose isotopic tracer analysis, showing that increased glucose carbons fuel phosphoenolpyruvate (PEP), pyruvate, and lactate in the resistant cells (Figure S1I-K). Next, to examine whether the resistant cells exhibit higher glucose dependency, we performed a cell viability assay in the presence of 2-deoxy-D-glucose (2-DG), a glucose analog that inhibits HK2 activity. Consistent with the ECAR and isotope tracing results, 2-DG remarkably reduced the growth of resistant cells, while no difference was observed in paired parental cells (Figure S1L-M). To determine whether the low OXPHOS resulted from FAO inhibition, we performed an OCR assay in the presence of palmitate by seahorse analysis. Indeed, compared to parental cells, both of those two resistant lines almost completely abolished FAO activity (Figure 1A-B). The SRC of OCR decreased by ~3.2-fold and ~2.5-fold in HepG2R cells and HuH7R cells, respectively (Figure 1C). In addition, the expression of peroxisome proliferator-activated receptor alpha (PPARα) and carnitine palmitoyltransferase 1 (CPT1), which are key FAO-related genes, significantly decreased in these resistant lines (Figure 1D). In contrast, HK2 expression increased in the resistant cells (Figure 1D). Moreover, in comparison to the parental cells, these two resistant lines were no longer sensitive to Etomoxir (ETO), a specific CPT1 inhibitor (Figure 1E). These observations above prompted us to examine whether intracellular FAs, which could not be normally metabolized by mitochondria, would be stored in LDs in sorafenib-resistant cells. Indeed, LD accumulation was clearly observed in HepG2R and HuH7R cells in the presence of either sorafenib or regorafenib (Figure 1F). Furthermore, sorafenib increased mitochondrial ROS generation in the parental cells, while a relatively low level of mitochondrial ROS maintained in the resistant cells (Figure 1G). Notably, peri-mitochondrial accumulation of LD was prominent in sorafenib-resistant cells, suggesting fatty acids fueling into TAGs for storage (Figure 1H). Next, to determine if the LD accumulation is critical for sorafenib resistance, we impaired LDs formation by using Triacsin C, an inhibitor of acyl-CoA synthetase (ACC). As expected, Triacsin C significantly induced PARP cleavage and inhibited cell growth in sorafenib-resistant HCC cells (Figure 1I, Figure S1N).

### Omics study identifies that AKR1C3 is responsible for TAG accumulation

**Figure 2:**
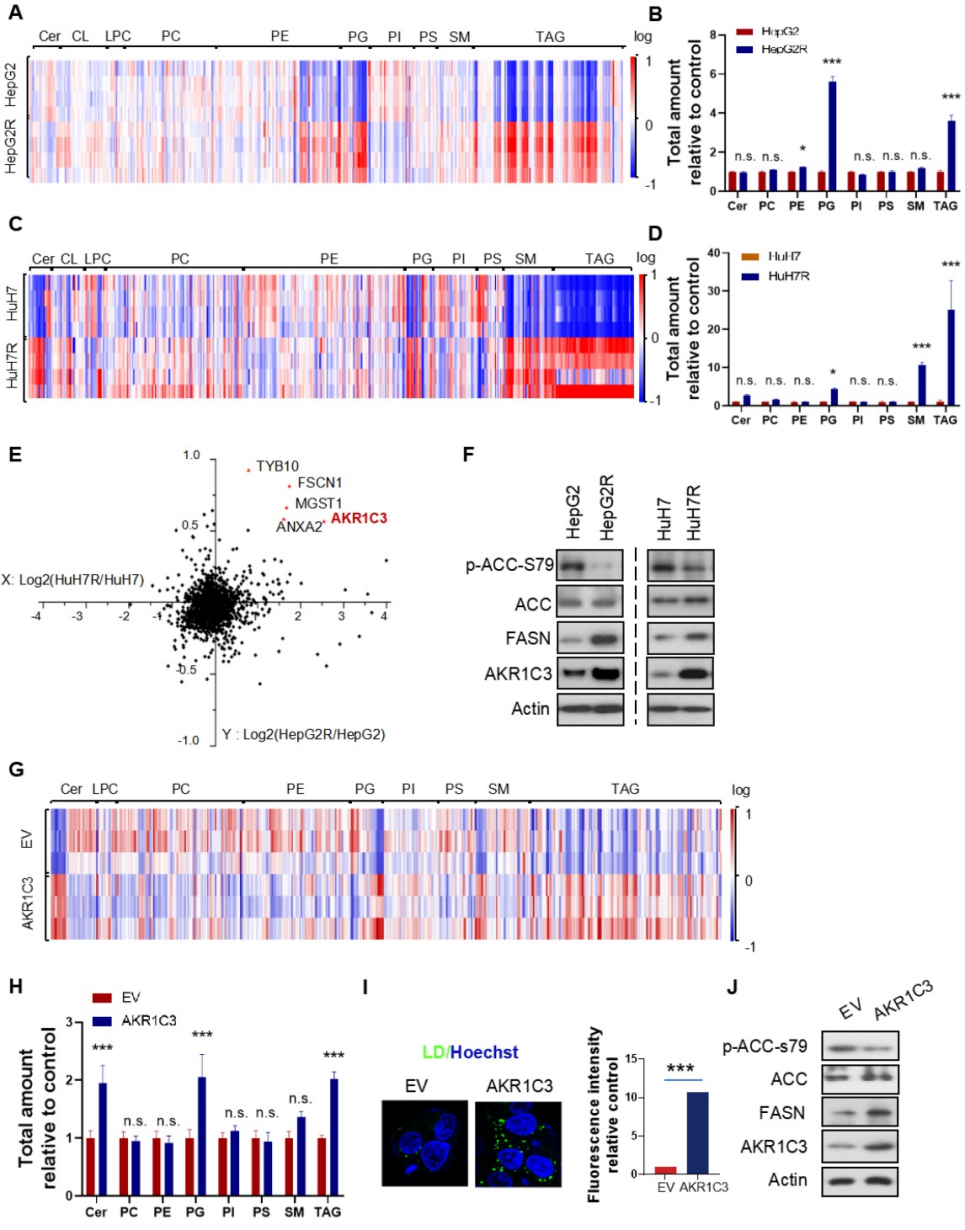
** Omics studies identified that increased AKR1C3 activity is critical for TAGs accumulation (A-D)** Heatmap and quantitative analysis of altered metabolites (FDR < 0.05) in two paired sorafenib resistant HCC cell lines. Relative abundance of each metabolite was quantified in both lines. **(E)** Logarithmic fold change of protein expression in sorafenib resistant cells compared to their parental cells. **(F)** The HepG2/HepG2R and HuH7/HuH7R cells were lysed and subjected to immunoblotting with the indicated antibodies. **(G-H)** Heatmap and quantitative analysis of altered metabolites (FDR < 0.05) in AKR1C3-overexpression HepG2 cells (HepG2-AKR1C3), in comparison to the empty vector-transfected cells (HepG2-EV). Relative abundance of each metabolite was quantified. P-values of each metabolite are shown on the top of graph. **(I)** LDs were stained using Bodipy493/503 (green) in both HepG2-EV and HepG2-AKR1C3 cells and analyzed by fluorescent microscopy. Nucleus was stained using Hoechst (blue).** (J)** HepG2-EV and HepG2-AKR1C3 cells were lysed and subjected to immunoblotting against the indicated antibodies. All data are quantified as mean ± SEM. Asterisk indicates significant difference. “n.s” indicates no significance, * p < 0.05, *** p < 0.001.

Next, to further investigate the metabolic features of the resistant cells, we performed a lipidomic analysis of both resistant and parental HCC cells. Consistent with the increased LD formation, we found that TAGs were the most significantly increased metabolites in resistant cells compared to their parental cells (Figure 2A&C). Besides TAGs, prostaglandins (PGs) also commonly increased in both of the resistant cell lines (Figure 2B&D). To identify the key protein responsible for lipid accumulation, we performed a proteomics assay in both parental and resistant cell lines. Interestingly, the expression of 5 proteins, including *HSD17B5* (*AKR1C3*)*, TYB10*,* MGST1*,* FSCN1,* and *ANXA2*, was commonly increased in both HepG2R and HuH7R lines compared with their parental lines (Figure 2E). Since it has been reported that the function of AKR1C3 is associated with lipid metabolism and lipogenesis in adipocytes of patients with polycystic ovary syndrome (PCOS) 28, we decided to investigate the role of this gene in lipid metabolism in HCC. The increased expression of AKR1C3 was validated by immunoblotting in both of the resistant HCC lines (Figure 2F). Moreover, consistent with the above results, expression of fatty acid synthase (FASN) and activity of acetyl-CoA carboxylase (ACC) increased in both resistant cell lines (Figure 2F). To further explore the potential role of AKR1C3 in lipid accumulation, we established the HepG2-AKR1C3 cell line stably overexpressing AKR1C3 for lipidomics analysis. Consistently, we showed that the metabolic signature of HepG2-AKR1C3 cells was quite similar to that of the resistant lines, characterized by the increased abundance of TAGs and PGs species (Figure 2G-H). In addition, overexpression of AKR1C3 promoted lipogenesis and LD production by decreasing the levels of p-ACC-s79 and increasing FASN expression compared to the empty vector-transfected line (Figure 2I-J), which is consistent with the observations in HepG2R cells. Together, these results suggest that AKR1C3 activity is critical for LD formation.

### Loss of AKR1C3 abrogates TAG accumulation and stimulates FAO

**Figure 3:**
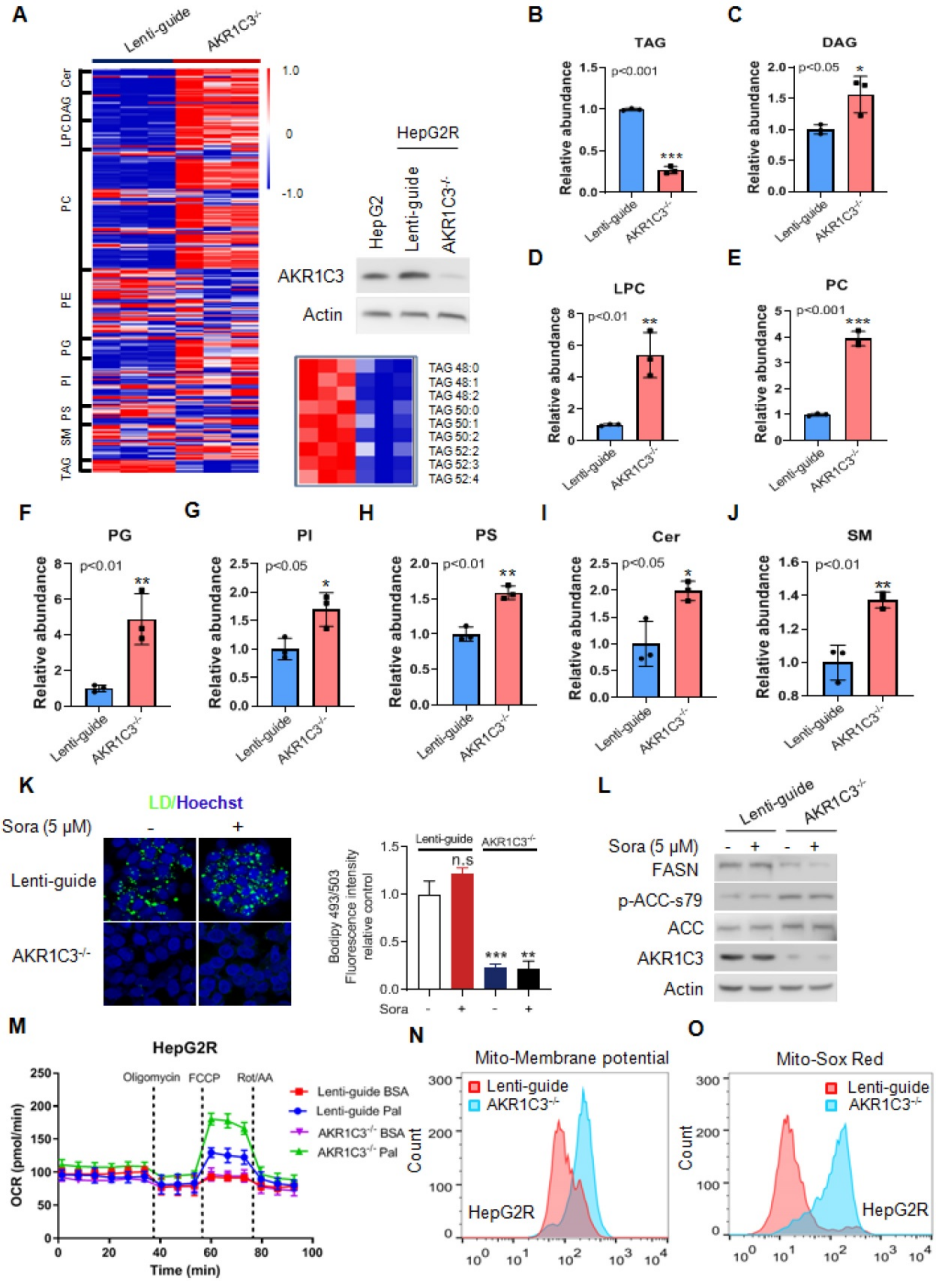
** Genetic deletion of AKR1C3 abrogates TAG accumulation in resistant HCC cells (A)** Heatmap showing the hierarchical clustering of the lipid species in the Lenti-guide control and AKR1C3^-/-^ groups, colored by abundance intensity. The efficacy of CRISPR/Cas9-based gene editing was evaluated by immunoblotting. Significant changes in TAGs species across those two groups were significant. **(B-J)** Relative abundance of significantly changed lipid species (Lenti-guide control and AKR1C3^-/-^ groups). **(K)** Lenti-guide control and AKR1C3^-/-^ HepG2R cells were treated with or without sorafenib for 24 h, then stained by Bodipy493/503. Cellular LD contents were visualized and evaluated by FACs and quantification of fluorescent intensity. Nucleus was stained using Hoechst (blue). **(L)** Lenti-guide control and AKR1C3^-/-^ HepG2R cells were treated with or without 5 μM sorafenib for 24 h, respectively. The total cell lysates were extracted and subjected to immunoblotting with indicated antibodies. **(M).** Oxygen consumption rates were measured in both Lenti-guide control and AKR1C3^-/-^ HepG2R cells incubated with BSA or palmitate, respectively. Oligomycin, FCCP, and rotenone/antimycin were added at the indicated time points.** (N-O)** Lenti-guide control and AKR1C3^-/-^ HepG2R cells were stained by Mito-tracker red CMXROS (red) and MitoSox (red) under basal conditions, respectively. The mitochondrial membrane potential was assessed by FACs assay and quantification of fluorescent intensity. All data are quantified as mean ± SEM. Asterisk indicates a significant difference and “n.s” indicates no significance. * p < 0.05, ** p < 0.01, *** p < 0.001.

Next, to determine if loss of AKR1C3 influenced the lipid accumulation in HCC, we knocked out AKR1C3 expression in HepG2R cells by CRISPR/Cas9-based gene editing, followed by lipidomic assay. We found that the most significantly altered metabolites were enriched in glycerophospholipid metabolism, glycerolipid metabolism, and glycosylphosphatidylinositol (GPI)-anchor biosynthesis pathway (Figure S2A-B). Moreover, loss of AKR1C3 markedly resulted in the depletion of TAGs, and up-regulation of the diacylglycerols (DAGs), glycerophospholipids (PC, LPC, PI, PS), and sphingolipid (Cer, SM) species (Figure 3A-J). Furthermore, TAGs depletion was also confirmed by immunofluorescence assay, showing the abolishment of LD formation in AKR1C3^-/-^ cells in comparison to the Lenti-guide control cells (Figure 3K, Figure S3A). In addition, immunoblotting showed that expression of FASN decreased whereas the level of p-ACC-s79 increased, suggesting that lipogenesis was inhibited in AKR1C3^-/-^ cells (Figure 3L, Figure S3B). Moreover, mitochondrial β-oxidation was enhanced in AKR1C3^-/-^ HepG2R cells, compared to the Lenti-guide control line (Figure 3M). Consistently, increased mitochondrial membrane potential and ROS generation were also clearly observed in AKR1C3^-/-^ cells (Figure 3N-O, Figure S3C-F). Taken together, these results strongly suggest that loss of AKR1C3 inhibits intracellular TAG accumulation, supporting the fatty acid transportation into mitochondria for β-oxidation.

### AKR1C3-dependent LD formation prevents mitochondrial dysfunction and lipotoxicity

**Figure 4:**
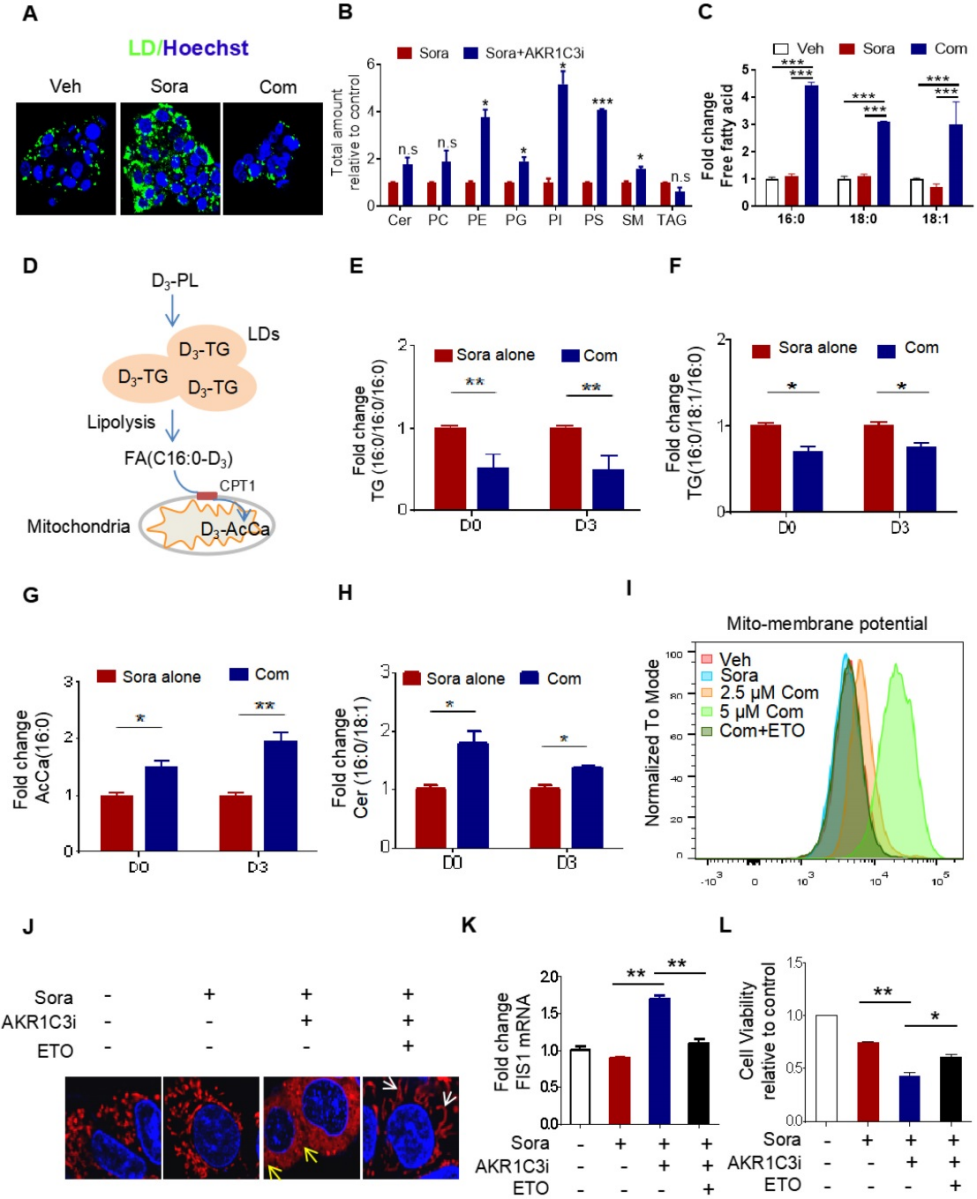
** AKR1C3 navigates FA flux into TAGs (A)** HepG2R cells were treated with vehicle or sorafenib, or combined with AKR1C3 inhibitor for 24 h, then stained using Bodipy493/503. Cellular LD contents were visualized by fluorescent microscopy. Nucleus was stained using Hoechst (blue). Com indicates the combination treatment of sorafenib with AKR1C3 inhibitor. **(B)** Quantitative analysis of altered metabolites (FDR < 0.05) in sorafenib-resistant HCC cell lines cultured with either sorafenib alone or sorafenib combined with AKR1C3 inhibitor. **(C)** Quantification showing the relative abundance of the intracellular free fatty acids (C16:0, C18:0, and C18:1).** (D)** Exogenous D3-PL transportation into LDs and fuel to mitochondria for β-oxidation. **(E-H)** HepG2 cells were incubated with either 0.5% BSA diluted d0-C16:0 or d3-C16:0 FFA. Cells were also treated with sorafenib or sorafenib combined with AKR1C3 inhibitor as indicated. Quantification showing the relative levels of lipids altered by AKR1C3 inhibitor. **(I-J)** HepG2R cells were treated with or without sorafenib or combined with AKR1C3 inhibitor and additional Etomoxir for 24 h. The cells were stained using MitoSox (red) followed by FACs assay. The cells were also stained by Mito-tracker red CMXROS (red) and visualized with fluorescence microscopy. Nucleus was stained using Hoechst (blue). Yellow arrow indicates the mitochondria undergoing fission, while the white arrow indicates fused or elongated mitochondria.** (K-L)** Relative mRNA level of FIS1 in HepG2R cells treated with or without sorafenib, sorafenib combined with AKR1C3 inhibitor, and additional Etomoxir for 24 h. Cell viability was assessed by Cell Titer-Glo assay. All data are quantified as mean ± SEM. Asterisk indicates significant difference. “n.s” indicates no significance,* p < 0.05, ** p < 0.01,*** p < 0.001.

It has been shown that LDs play a crucial role in reducing mitochondrial lipotoxicity for cell survival by regulating the mobilization of FAs 10, 29. Based on our data, we hypothesize that AKR1C3 promotes LD formation by inhibiting LD degradation. Because there was accumulated LD in AKR1C3^-/-^ cells, we attempted to use specific inhibitors against AKR1C3 to dynamically investigate whether inhibition of AKR1C3 induces LD breakdown. HepG2R cells were treated with flufenamic acid (FLU) or 5-β cholanic acid (5-β CA), two small molecules inhibiting AKR1C3 activity, then subjected to lipidomic analysis. Consistent with the observation in AKR1C3^-/-^ cells, AKR1C3 inhibition by either of these two inhibitors significantly decreased the level of TAGs, and depleted LD contents in the presence of sorafenib (Figure 4A, Figure S4A-C), while the levels of phospholipid and sphingolipid species increased, including PE, PG, PI, PS and SM (Figure 4B). Since long-chain saturated FAs (C16:0) and monounsaturated FAs (C18:1) are mainly esterified and transported into mitochondria by CPT1 for β-oxidation 30, 31, we investigated the distribution of these fatty acids between TAGs and intracellular free fatty acids (FFAs). Consistently, we found that AKR1C3 inhibition remarkably reduced the abundance of both C16:0-TAG and C18:1-TAG, while it increased the intracellular levels of C16:0-FA, C18:0-FA, and C18:1-FA (Figure 4C, Figure S4D). To specifically track FAs flux, we performed isotopic tracing analysis using deuterium3-labeled palmitate (D3-PL) (Figure 4D). The incorporation of D3-FFA into lipids was measured by LC-MS/MS-based analysis. As expected, AKR1C3 inhibition reduced D3-PL incorporation into C16:0-TAGs species, including TAGs (C16:0/C16:0/C16:0) and TAGs (C16:0/C18:1/C16:0), while it induced the accumulation of D3-PL-acylcarnitine (~2-fold) and D3-Cer (~1.5 fold) in FLU-treated cells (Figure 4E-H). To further confirm the effect of AKR1C3 inhibition on mitochondrial function, we performed OCR assays in the resistant cells. Consistent with our results, FLU treatment fully impaired mitochondrial SRC in both resistant lines upon treatment of sorafenib (Figure S4E-F). Moreover, this combinatorial treatment abnormally increased mitochondrial membrane potential by ~2-fold, which was effectively rescued by additional treatment of ETO (Figure 4I, Figure S4G). In addition, pharmacologic inhibition of AKR1C3 increased mitochondrial ROS generation, and markedly triggered mitochondrial fission protein 1 (FIS1)-related mitochondrial fission and apoptosis in HepG2R cells (Figure 4J-K, Figure S4H-I). Similarly, ETO treatment promoted mitochondrial elongation/fusion and increased cell viability (Figure 4J-L).

### AKR1C3 inhibits lipophagy to support LD formation

**Figure 5:**
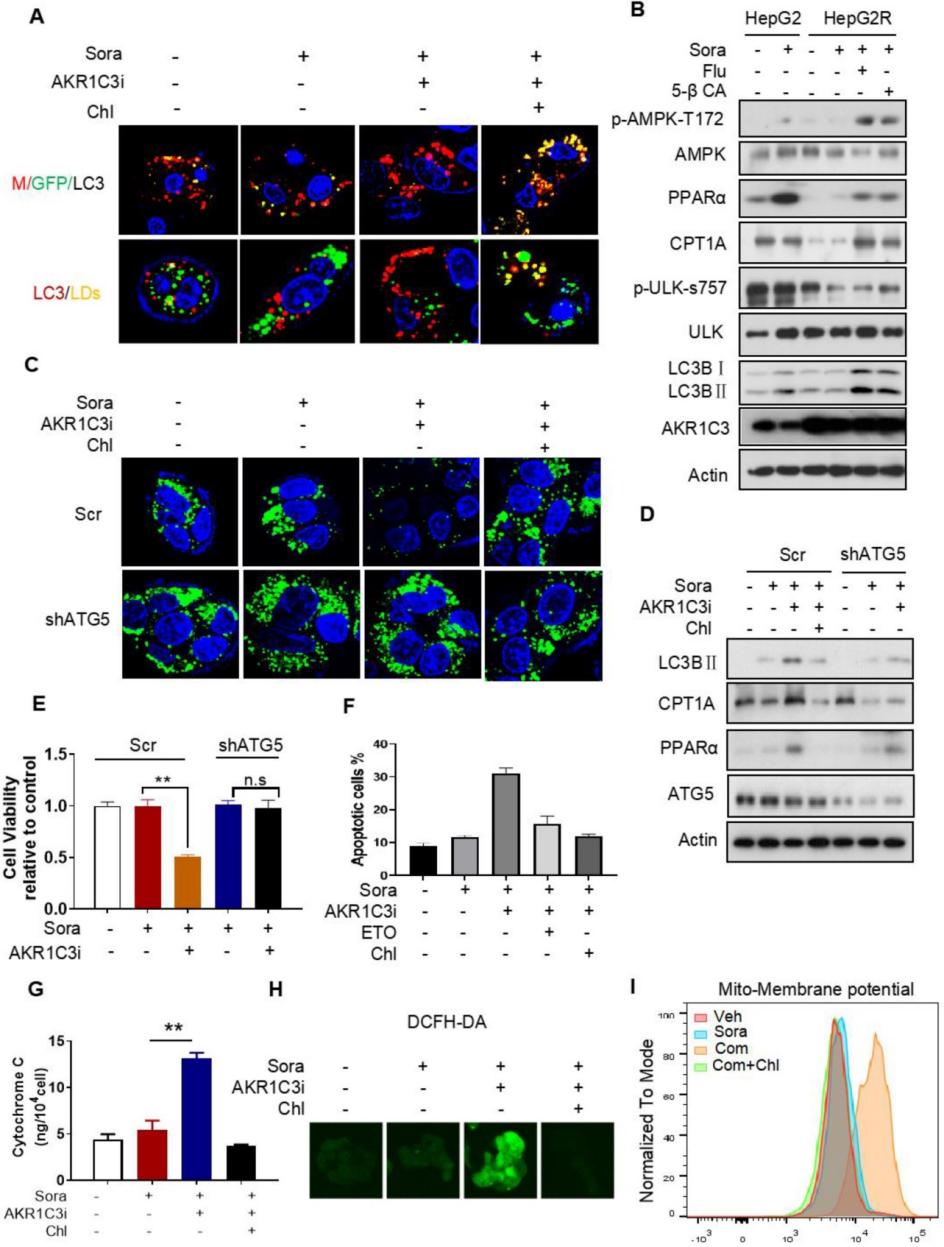
** AKR1C3 inhibits autophagy-dependent LD degradation (A)** Empty vector and mCherry-GFP-LC3-transfected HepG2R cells were incubated with vehicle or 5 μM sorafenib or combined with AKR1C3 inhibitor and additional chloroquine for 24 h. Autophagy flux (above) and co-localization (below) of LDs (green) and LC3 (red) were evaluated by immunofluorescence staining. Representative images are shown.** (B)** HepG2 and HepG2R cells were treated with vehicle or sorafenib or combined with AKR1C3 inhibitors (FLU or 5β-CA) for 24 h. The cells were lysed and subjected to immunoblotting against the indicated antibodies.** (C)** HepG2R-Scr and HepG2R-shATG5 cells were treated with vehicle or sorafenib or combined with AKR1C3 inhibitor and additional Chloquine for 24 h. The LDs and nucleus were stained using Bodipy (green) and Hoechst (blue), respectively. Representative images were shown. **(D)** HepG2R-Scr and HepG2R-shATG5 were incubated with vehicle or sorafenib or sorafenib combined with AKR1C3 inhibitor and additional chloroquine for 24 h. The cells were lysed and subjected to immunoblotting against the indicated antibodies. **(E)** HepG2R-Scr and HepG2R-shATG5 cells were incubated with vehicle or sorafenib or sorafenib combined with AKR1C3 inhibitor for 24 h. Cell viability was assessed by Cell Titer-Glo assay. **(F)** HepG2R cells were incubated with vehicle or sorafenib or sorafenib combined with AKR1C3 inhibitor. These HepG2R cells were additionally treated with etomoxir or chloroquine. Cellular apoptosis was evaluated by crystal violet staining and Annexin V/PI double staining. The percentage of apoptotic cells was quantified by FACs and fluorescent intensity. **(G)** HepG2R cells were incubated with sorafenib or combined with AKR1C3 inhibitor and additional chloroquine for 8 h. Intracellular cytochrome C was measured. **(H)** HepG2R cells were treated with or without sorafenib or combined with FLU and additional chloroquine. Total cellular ROS were assessed by DCFH-DA staining and subjected to immunofluorescence microscopy. Representative images are shown.** (I)** HepG2R cells were treated with either vehicle or sorafenib or sorafenib combined with AKR1C3 inhibitor and additional chloroquine for 24 h. The cells were stained using Mito-tracker red CMXROS (red), then subjected to FACs assay. Com represents sorafenib combined with AKR1C3 inhibitor. All data are quantified as mean ± SEM. Asterisk indicates significant difference, “n.s” indicates no significance, ** p < 0.01.

Next, we investigated how AKR1C3 regulates lipid accumulation. Since the basic function of AKR1C3 is to regulate PG biosynthesis 32, 33, we first determined if PG metabolism contributes to LD accumulation in HCC. We treated HCC cells with PGD_2_, a direct substrate of AKR1C3, and showed that exogenous PGD_2_ increased neither cell viability nor the number of LDs upon treatment with sorafenib in the parental HCC cells (Figure S5A-D). Conversely, inhibition of PGs metabolism and action by aspirin (COX1/2 inhibitor), celecoxib (COX2 inhibitor), or PF04418948 (an antagonist of PGE_2_ receptor) did not sensitize resistant cells to sorafenib treatment (Figure S5E-F). These data suggest that AKR1C3 mediates sorafenib resistance and lipid accumulation in a PG-independent manner. It has been shown that inhibition of AKR1C3 in these cells enhanced doxorubicin (DOX)-elicited autophagy in gastrointestinal cancer cell lines 34. Thus, we hypothesized that AKR1C3 may regulate LD homeostasis via autophagy-mediated LD breakdown (lipophagy). To determine whether AKR1C3 inhibition triggered autophagy-dependent LD breakdown, a tandem mCherry-GFP-LC3 fusion protein assay was utilized to assess autophagic flux in HepG2R cells. As expected, inhibition of AKR1C3 by FLU markedly reduced GFP levels, which could be effectively diminished by chloroquine (Chl), a commonly used inhibitor of autophagy (Figure 5A). Similarly, using LC3B-overexpressing HepG2R cells, we found that AKR1C3 inhibition significantly eliminated LD accumulation, while additional treatment of Chl increased the number of LC3B-positive autophagosomes and enriched LC3B positivity around and within LDs (Figure 5A). In addition, inhibition of AKR1C3 by either FLU or 5-β CA significantly induced the levels of phospho-adenosine 5-monophosphate (AMP)-activated protein kinase (AMPK)-T172 and LC3B cleavage while decreasing the level of phospho-unc-51-like-kinase1 (ULK1)-S757, an inhibitory site of ULK1, in response to sorafenib treatment (Figure 5B, Figure S6A). Furthermore, the combined treatment restored the expression of CPT1A and PPARα, promoting nuclear translocalization of PPARα in HepG2R cells (Figure 5B, Figure S6A-B). To further examine whether LD breakdown by AKR1C3 inhibition is dependent on autophagy, we knocked down the expression of autophagy-related gene5 (ATG5), which is a key molecule mediating autophagosome formation. Consistently, knockdown of ATG5 abrogated AKR1C3 inhibitor-induced LD clearance, which was similar to the effect of Chl treatment (Figure 5C). Concomitantly, loss of ATG5 suppressed AKR1C3 inhibitor-induced LC3 cleavage, restoring PPARα and CPT1A expression in the presence of sorafenib (Figure 5D). These findings prompted us to examine whether inhibition of autophagy/lipophagy enhances the viability and suppresses apoptosis of HCC cells upon the combined treatment. In agreement with our above results, either Chl or knockdown of ATG5 markedly enhanced cell survival and decreased the population of annexin V+/PI+ cells upon the combined treatment (Figure 5E-F, Figure S6C). Finally, Chl also markedly decreased FLU-induced cytochrome C release, ROS generation, and increased LDs in the presence of sorafenib (Figure 5G-H, Figure S6D). Notably, increased mitochondrial membrane potential by co-treatment of sorafenib and FLU could shift back to the normal range with additional treatment of Chl (Figure 5I). Taken together, these results suggest that AKR1C3 regulates LD homeostasis in HCC cells through lipophagy.

### AKR1C3 reprograms the metabolic switch from OXPHOS to glycolysis

**Figure 6:**
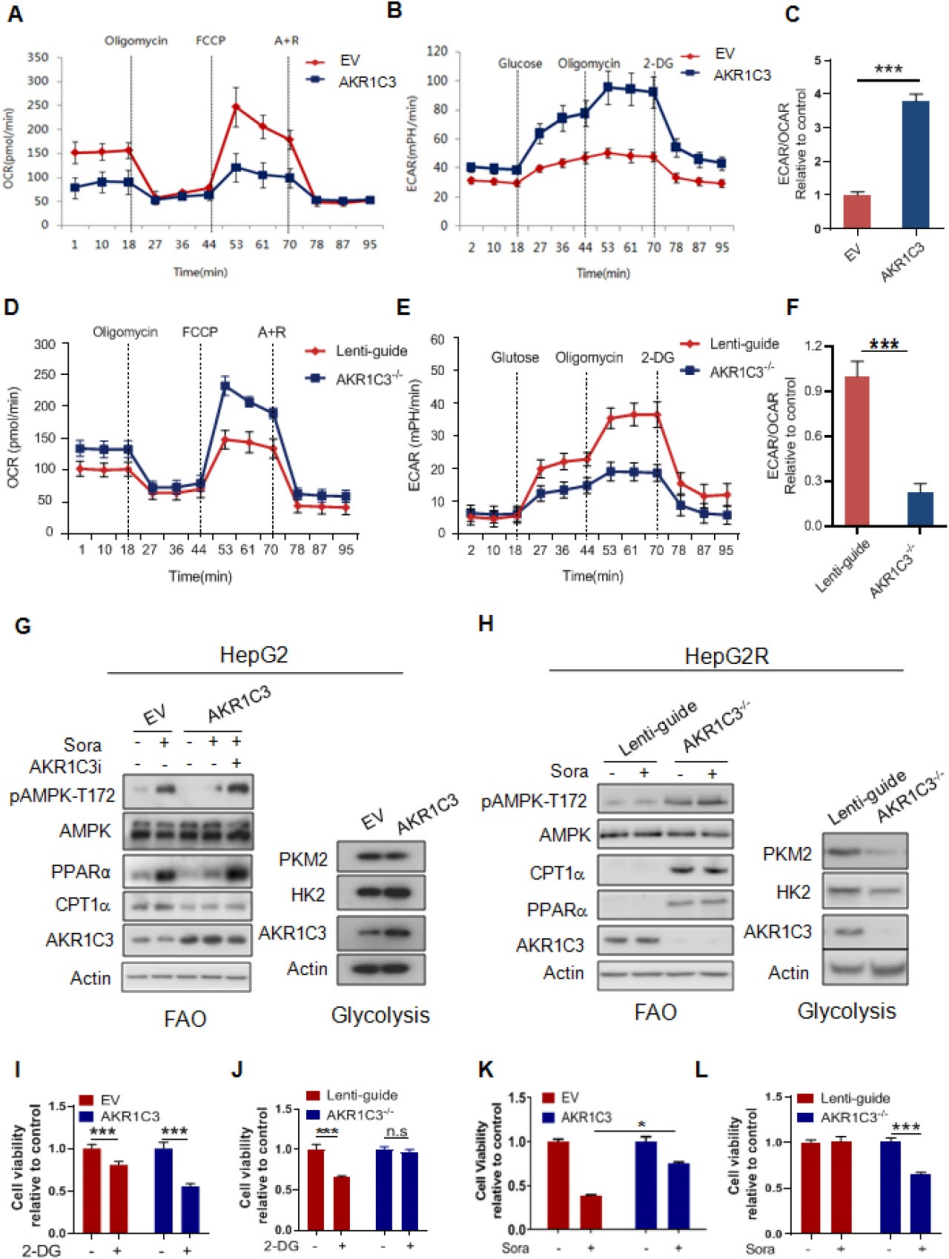
** AKR1C3 controls metabolic balancing between FAO and glycolysis (A)** Oxygen consumption rate was measured by Seahorse XF-96 assay in HepG2-EV and HepG2-AKR1C3 cells. Oligomycin, FCCP, antimycin, and rotenone were added at the indicated time points. **(B)** Extracellular acidification rates were measured by Seahorse XF-96 assays in HepG2-EV and HepG2-AKR1C3 cells. Glucose, Oligomycin, and 2-DG were added at the indicated time points. **(C)** Ratios of ECAR to OCR in HepG2-EV and HepG2-AKR1C3 cells. **(D)** Oxygen consumption rates were measured by Seahorse XF-96 assays in Lenti-guide control and AKR1C3^-/-^ HepG2R cells. Oligomycin, FCCP, antimycin, and rotenone were added at the indicated time points. **(E)** Extracellular acidification rate was measured by Seahorse XF-96 assays in Lenti-guide and AKR1C3^-/-^ HepG2R cells. Glucose, oligomycin, and 2-DG were added at the indicated time points. **(F)** Ratios of ECAR to OCR in Lenti-guide control and AKR1C3^-/-^ HepG2R cells. **(G)** HepG2-EV and HepG2-AKR1C3 cells were cultured with or without sorafenib or combined with AKR1C3 inhibitor for 24 h. The extracted proteins were analyzed by immunoblotting with the indicated antibodies. **(H)** Lenti-guide control and AKR1C3^-/-^ cells HepG2R cells were incubated with or without sorafenib or combined with AKR1C3 inhibitor for 24 h. The extracted protein was analyzed by immunoblotting against the indicated antibodies. **(I-J)** HepG2-EV, HepG2-AKR1C3, Lenti-guide control, and AKR1C3^-/-^ HepG2R cells were treated with or without 2-DG for 24 h. Cell viability was assessed by Cell Titer-Glo assay. **(K-L)** HepG2-EV, HepG2-AKR1C3, Lenti-guide control, and AKR1C3^-/-^ HepG2R cells were treated sorafenib for 24 h. Cell viability was assessed by Cell Titer-Glo assay. All data are quantified as mean ± SEM. Asterisk indicates significant difference, “n.s” indicates no significance, * p < 0.05, *** p < 0.001.

Our results suggest that AKR1C3 promotes intracellular lipid storage and inhibits fatty acid oxidation, thus protecting HCC cells from acylcarnitine-induced mitochondrial dysfunction. This is consistent with the findings that sorafenib-resistant cells rely on glycolysis, rather than FAO/OXPHOS, for energy production to reduce mitochondrial ROS generation. To determine whether AKR1C3 activity affects energetic metabolism, we modulated the levels of AKR1C3 via either overexpression in parental HepG2 cells or CRISPR/Cas9-based genetic deletion in sorafenib-resistant HepG2 cells. Interestingly, overexpression of AKR1C3 significantly decreased the OCR but increased the ECAR in their parental cells (Figure 6A-B). Furthermore, the ratio of ECAR/OCR increased ~3.7-fold (Figure 6C). In contrast, deletion of AKR1C3 reduced ECAR but increased OCR (Figure 6D-E). The ratio of ECAR/OCR decreased by ~2-fold in AKR1C3^-/-^ cells relative to the control (Figure 6F). Moreover, overexpression of AKR1C3 decreased the levels of p-AMPK-T172, PPARα, and CPT1A, but increased the protein levels of HK2 (Figure 6G). Notably, AKR1C3 inhibition by FLU restored the levels of the above proteins in sorafenib-treated HCC cells (Figure 6G). In contrast, depletion of AKR1C3 increased the levels of p-AMPK-T172, PPARα, and CPT1A, but decreased the protein levels of HK2 and PKM2 in sorafenib-resistant cells (Figure 6H). Furthermore, AKR1C3-overexpressing HepG2 cells exhibited more growth dependency on glucose since treatment of 2-DG significantly reduced cell viability, in comparison to the empty vector-transfected control cells (Figure 6I). Conversely, AKR1C3^-/-^ HepG2R cells were less sensitive to 2-DG compared to Lenti-guide control HepG2R cells (Figure 6J). Finally, overexpression of AKR1C3 markedly enhanced cell survival in HepG2 cells in the presence of sorafenib, while deletion of AKR1C3 sensitized HepG2R cells to sorafenib (Figure 6K-L). Collectively, these results suggest that AKR1C3 drives the metabolic shift from FAO towards glycolysis to facilitate sorafenib resistance in HCC cells.

### AKR1C3 sustains HCC cell growth upon sorafenib treatment *in vivo*

**Figure 7:**
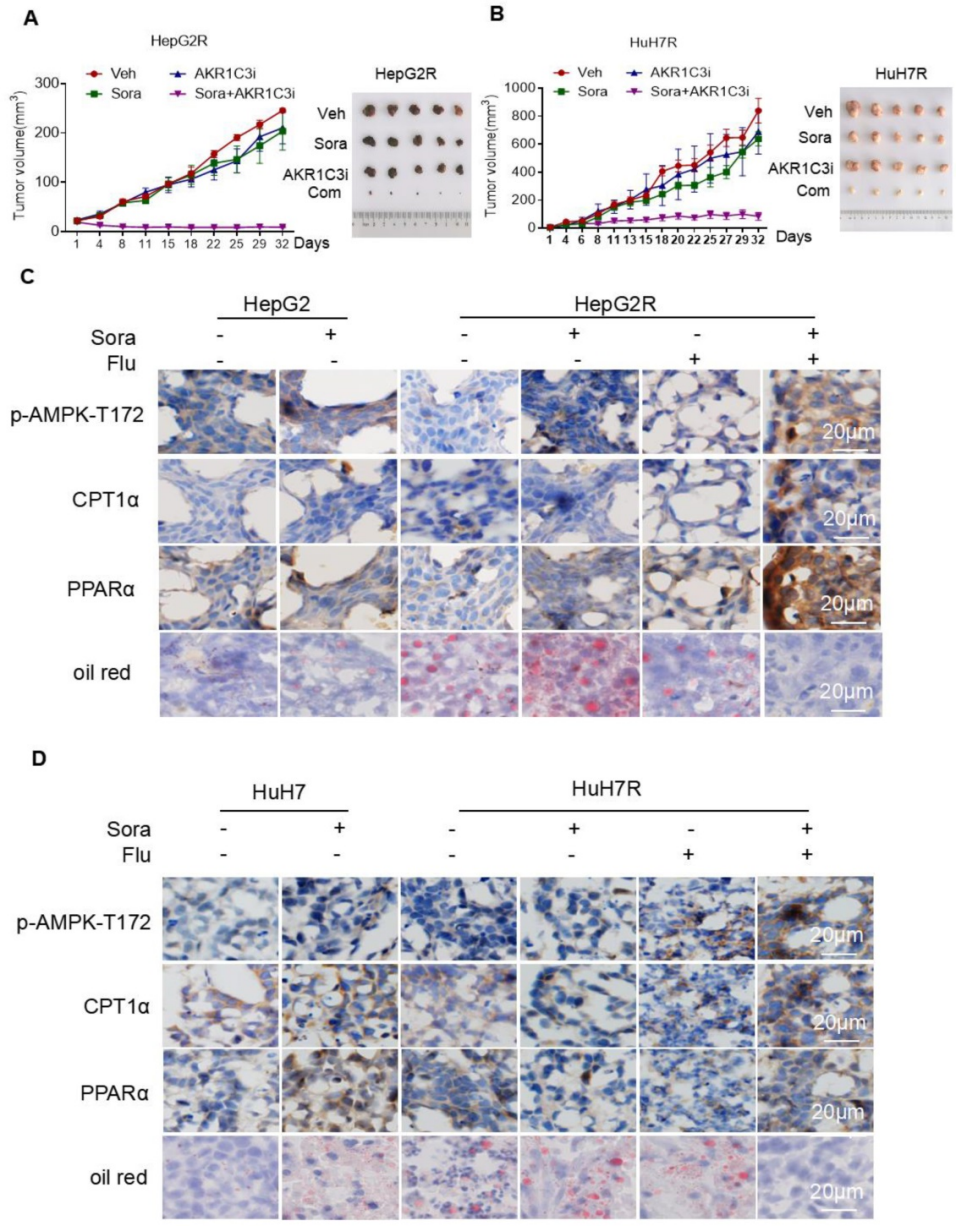
** Inhibition of AKR1C3 leads to sorafenib-resistant HCC tumor regression in xenograft mice (A-B)** HepG2/HepG2R and HuH7/HuH7R cells were injected into BALB/c nude mice. Tumor volume was calculated using caliper measurements (π/6 × length × width^2^). n = 5 for each group. Once the subcutaneous tumors reached the volume of 300 mm^3^, the treatment began. Mice were randomly subjected to the vehicle, sorafenib (25 mg/kg), flufenamic acid (25 mg/kg), or a combination of sorafenib. Flufenamic acid was administered every 2-3 days at the same dose. Four weeks post-implantation, tumors were isolated from each group. Figures of isolated tumor are shown on the right. **(C-D)** Xenograft tumors from each group were sectioned and subjected to immunohistochemistry against the indicated antibodies. Oil red was used to stain lipid droplets in these tumors. Scale bar, 20 μm. The mice were randomly selected from each group. Representative images are shown.

To investigate whether AKR1C3-mediated LDs accumulation potentiates the vulnerability of sorafenib-resistant HCC cells, we examined the effect of the combined treatment of sorafenib with FLU on tumor growth in a xenograft model. Consistent with our *in vitro* results, the combination of an AKR1C3 inhibitor with sorafenib strongly suppressed the growth of HepG2R and HuH7R xenograft tumors compared to the sorafenib alone group (Figure 7A-B). To further investigate whether the resistant cells were undergoing LDs degradation, we performed immunohistochemistry to assess the expression of CPT1A and PPARα. As expected, we found that the expression of these two proteins was significantly elevated in xenograft tumors upon the combined treatment (Figure 7C-D). Moreover, we also evaluated the LDs status in these tumors by oil-red staining. In agreement with our findings, the combined treatment abolished LDs accumulation in xenograft tumors of HCC cells (Figure 7C-D). To further confirmed the role of AKR1C3 in lipid metabolism and sorafenib resistance, we subcutaneously injected Lenti-guide control and AKR1C3^-/-^ HepG2R cells in our mice model. Consistently, loss of AKR1C3 synergized with sorafenib to inhibit tumor growth by inducing lipophagy (Figure S7A-C). Collectively, our data indicate that inhibition of AKR1C3 sensitized the resistant HCC cells to sorafenib by inducing LD breakdown and mitochondrial lipotoxicity in the xenograft tumor model of HCC.

## Discussion

Cancer cells can dynamically shift their relative reliance on glycolytic versus oxidative metabolism in response to nutrient depletion, hypoxia, and drug treatment 35-37. Liver cancer cells treated with sorafenib have recently been characterized by increased glycolytic flux and deceased mitochondrial OXPHOS in both HCC patients and cultured cell lines 3, 6. In this study, we demonstrated that long-term sorafenib-treated HCC cells relied on glycolysis, rather than FAO/OXPHOS for energy production and survival. The ablation of FAO was supported by decreased PPARα and CPT1A expression, as well as the fact that the resistant cells were no longer sensitive to ETO. These observations may not be surprising due to sorafenib inhibition of the activity of complex II/III of the electron transport chain (ETC) and ATP synthase 4. Of note, HCC tumor cells, especially those with activating CTNNB1 mutations, highly rely on FAO for proliferation and survival 7, leading us to wonder how the resistant cells deal with the excess intracellular FAs when the fatty acid catabolism is inhibited. Thus, we speculated that increased lipid storage might be required to mitigate the lipotoxicity upon sorafenib treatment. Indeed, LD accumulation was clearly observed in both sorafenib-resistant cell lines in the presence of sorafenib.

Although the increased LD formation has been reported in HCC, its function and regulatory mechanism remain unclear in liver cancer. Recent studies have shown that HSD17B13, a member of the 17β-hydroxysteroid dehydrogenase family, plays a critical role in the pathogenesis of nonalcoholic fatty liver disease (NAFLD) 38, indicating a role of this family in regulating hepatic lipid metabolism. Using a multi-omics-based assay, we identified increased expression of HSD17B5/AKR1C3, another member of this family, in sorafenib-resistant HCC cells. Of note, it has been shown that sorafenib can increase Nrf2 activation in HCC cells 39. Furthermore, AKR1C3 has been reported to be one of the downstream targets of Nrf2 40. Therefore, it could be speculated that sustained Nrf2 activation may contribute to the elevation of AKR1C3 in sorafenib-resistant HCC tumor cells.

It has been shown that AKR1C3 expression is associated with lipogenesis in patients with PCOS 28, leading us to examine if AKR1C3 activity is critical for lipid metabolism in HCC. This was supported by the observations that overexpression of AKR1C3 increased TAGs abundance, suggesting that AKR1C3 activity may navigate FAs flux into TAGs. Consistently, genetic deletion of AKR1C3 completely abolished TAG accumulation, promoting FAO and mitochondrial ROS generation. These results suggest that inhibition of AKR1C3 activity may induce LD breakdown. Using deuterium^3^-labeled palmitate tracer analysis, we confirmed that AKR1C3 inhibition significantly decreased the abundance of D3-TAGs, especially for FA (16:0/16:0/16:0) and FA (16:0/18:1/16:0) species, which could be esterified and transported into the mitochondria for β-oxidation. In addition, inhibition of AKR1C3 resulted in the accumulation of AcCa (16:0) and Cers (16:0/18:1). In this context, this process led to severe mitochondrial dysfunction, which was further confirmed by complete loss of mitochondrial respiration capacity and abnormal mitochondrial membrane potential, as well as high levels of mitochondrial ROS generation. Notably, the combined treatment induced FIS1-related mitochondrial fission, which could be completely rescued by ETO. Since mitochondrial fission likely determines cell death 41, we showed that inhibition of AKR1C3 significantly induced apoptosis, which could be also rescued by ETO. These findings suggested that LD production by AKR1C3 activity is required to protect HCC cells from mitochondrial dysfunction, maintaining a low level of mitochondrial ROS generation.

**Figure 8:**
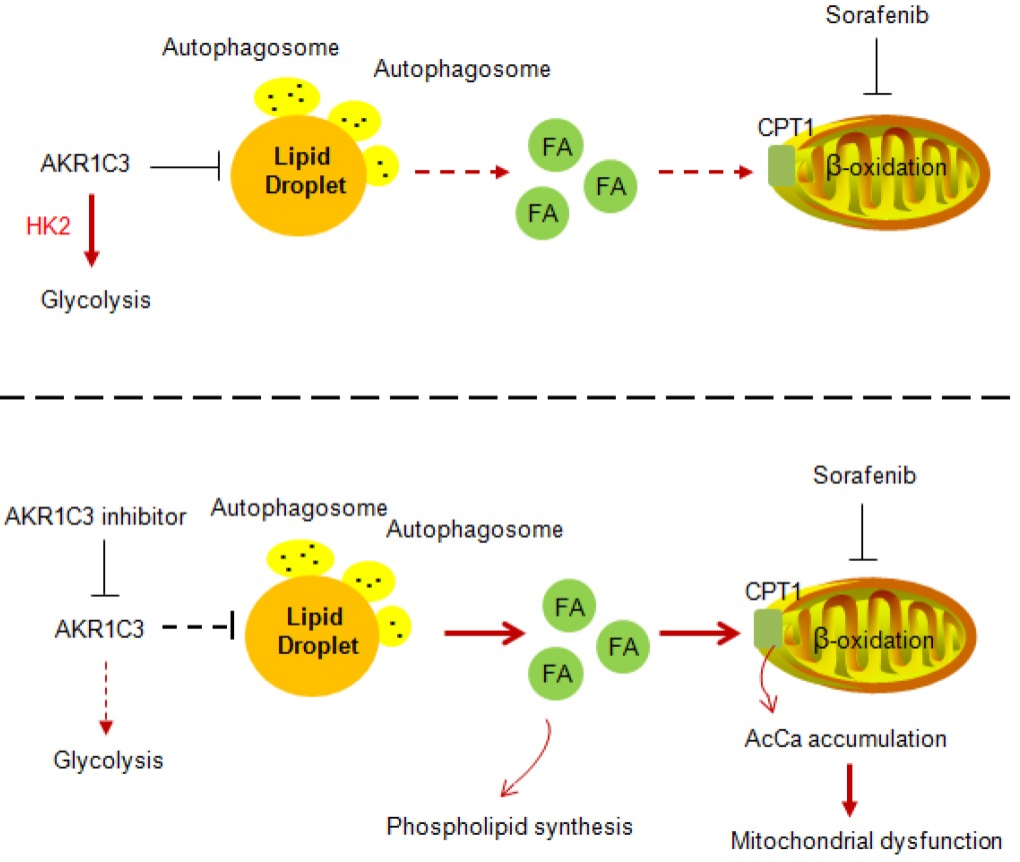
Proposed model of AKR1C3-dependent LD metabolism in mitigating lipotoxicity in sorafenib-resistant HCC.

Our data strongly imply a role of AKR1C3 in regulating LD homeostasis. The basic biological function of AKR1C3 is to catalyze the reduction of prostaglandin (PG) D2 and PGH2, and the oxidation of 9α, 11β-PGF2 to PGD2. In addition, a recent study showed that combined treatment of sorafenib with flufenamic acid could enhance the anti-tumor effect of sorafenib, likely by inhibiting PG metabolism 42. However, our data clearly showed that lipid accumulation by AKR1C3 was completely independent of PG metabolism because either treatment with PGD2 or pharmacologic inhibitors of the PG metabolic pathway did not affect sorafenib sensitivity and lipid accumulation in HCC cells. Another study showed that the knockdown of AKR1C3 overcame doxorubicin resistance by promoting autophagy in gastrointestinal cancer cells 34. In addition, FLU, an AKR1C3 inhibitor, has been reported to activate AMPK signaling, which directly regulates autophagy via ULK1 phosphorylation 43, 44. These findings led us to examine whether LD accumulation by ARK1C3 depends on autophagy. The hypothesis was primarily supported by the colocalization of the AKR1C3 protein with lysosomes. Moreover, AKR1C3 inhibition activated AMPK, leading to increased LC3 cleavage and reduced phosphorylation of the ULK1 inhibitory site. Importantly, the induction of lipophagy by AKR1C3 inhibition was further confirmed by classic LC3/mCherry/GFP transfection assay and LC3/LDs co-staining. Meanwhile, either loss of ATG5 or chloroquine treatment completely abolished lipophagy induced by the AKR1C3 inhibitor, maintaining low levels of mitochondrial ROS, and cell survival. Taken together, these findings revealed that AKR1C3 regulates lipid homeostasis through lipophagy (Figure 8). A recent study showed that STAT3 directly binds to the AKR1C3 promoter, regulating the transcription of AKR1C3 in HCC 45. In addition, STAT3 signal regulates hepatic LD accumulation in diabetic mice and promotes the “Warburg effect” in liver cancer 46-48. These results are consistent with our conclusion that AKR1C3 is a key regulator in hepatic glucose and lipid metabolisms. Further study may be needed to address the importance of AKR1C3 in hepatic lipid metabolism.

Our data also showed that AKR1C3 inhibition restored the expression of PPARα and CPT1A in the presence of sorafenib in resistant HCC cells. An interpretation could be that lipophagy induced by AKR1C3 inhibition triggered the release of cytosolic free fatty acids, resulting in PPARα activation. Indeed, inhibition of lipophagy by Chl significantly decreased the expression of PPARα and CPT1A. These observations also raised the question of whether the expression of AKR1C3 can directly impact energy metabolism. Interestingly, we demonstrated that manipulation of AKR1C3 expression could affect the metabolic balance between glycolysis and FAO. It has been recently reported that AKR1B10, another member of the aldo-keto family reductases, promotes the metabolic shift from glycolysis towards FAO, supporting the metastasis of breast cancer cells 49. In this study, we demonstrated that AKR1C3 drives FAO towards glycolysis in support of cell survival under sorafenib treatment. Since both AKR1C3 and AKR1B10 were elevated in liver tumor tissues 50, an interplay between AKR1B10 and AKR1C3 by balancing FAO and glycolysis might be required to adapt to differential tumor environments and metabolic demands.

In summary, we demonstrated that AKR1C3 is required to mitigate cellular lipotoxicity by promoting LD formation in a lipophagy-dependent manner in HCC tumors. These findings raise the possibility that AKR1C3-dependent LD metabolism is more widely involved as a protective lipid buffering system in the liver under cellular stress conditions, including high-fat diet (HFD), hypoxia, and nutrient depletion. Future research exploring the mechanism of lipid metabolism reprogrammed by AKR1C3 will be required to understand the pathogenesis of aberrant lipid metabolism-associated liver cancer, such as nonalcoholic steatohepatitis (NASH) driven-HCC.

## Materials and Methods

### Ethics statement

All animal procedures were performed in accordance with the NIH Guide on the Care and Use of Laboratory Animals and approved by the Institutional Animal Care and Use Committee of the China Medical University.

### Cell Culture

All the HCC cell lines were purchased from ATCC Inc. and cultured in DMEM containing 4.5 g/L glucose and L-glutamine (12100046, Gibco, China) supplemented with 10% fetal bovine serum (10100147, Gibco, Australia) at 37 °C and 5% CO_2_.

### Lipid Droplet Staining

Cells grown on glass-bottom dishes (NEST, WuXi, China) were incubated with 5 μM sorafenib (S7397, Selleck), 20 μM flufenamic acid (530-78-9, Sigma), or 50 μM 5-β cholanic acid (1553-56-6, Sigma) for 24 h. After washing with PBS, cells were stained using BODIPY 493/503 (D3922, Thermo Scientific™), while the nuclei were stained by Hoechst 33342 (62249, Thermo Scientific™). Cells were washed with cold PBS and subsequently subjected to confocal microscopy.

### Immunoblotting

Cells were lysed in RIPA lysis buffer (Beyotime, Shanghai, China) for 0.5 h at 4 °C, loaded on 10-12% SDS-PAGE gels, and transferred onto a PVDF (IPVH00010, Millipore) membrane. The membranes were washed by 1×TBS plus 0.1% Tween-20 (TBST) and blocked in TBST containing 5% defatted milk for 1 h. Membranes were incubated for at least 12 h in TBST containing 5% BSA and primary antibody at 4 °C. The antibodies used in this study were as follows: rabbit anti-PKM2 (4053, CELL SIGNALING), rabbit anti-Hexokinase 2 (22019-1-AP, Proteintech), rabbit anti-PPARα (15540-1-AP, Proteintech), rabbit anti-CPT1A (12252, CELL SIGNALING), rabbit anti-Phospho-Acetyl-CoA Carboxylase-Ser79 (11818, CELL SIGNALING), rabbit anti-Acetyl CoA Carboxylase (3662, CELL SIGNALING), rabbit anti-Fatty Acid Synthase (3180, CELL SIGNALING), rabbit anti-AKR1C3 (1:1000, PA5-28065, ThermoFisher), rabbit anti-pAMPKα-Thr172(2535, CELL SIGNALING), rabbit anti-AMPKα (2532, CELL SIGNALING), rabbit anti-Phospho-ULK1-Ser638 (14205, CELL SIGNALING), rabbit anti-LC3A/B (12741, CELL SIGNALING), rabbit anti-ATG5 (12994, CELL SIGNALING), and mouse anti-β-actin (sc-47778, Santa Cruz). After washing with TBST 3 times, the membranes were incubated with HRP-conjugated secondary antibodies diluted in 5% TBST at room temperature for 1 h. All membranes were exposed to X-Ray film by electrochemiluminescence (ECL) (180-501, Tanon, Shanghai, China).

### Immunohistochemistry

Tumor samples were embedded in paraffin, dewaxed, then rehydrated in 95% alcohol. Further antigen retrieval process was performed before the incubation with primary antibody. After washing in TBST, the secondary antibody was added to the slide and incubated at room temperature for 1 h. Slides were stained with hematoxylin and eosin (H&E). Images were captured by Olympus microscopy CX41 (Tokyo, Japan). The following primary antibodies were used: rabbit anti-Phospho-Acetyl-CoA Carboxylase (Ser79) (1:1000, 11818, CELL SIGNALING), rabbit anti-PPARα (15540-1-AP, Proteintech), rabbit anti-CPT1A (1:2000, ab234111, Abcam), and rabbit anti-AKR1C3 (PA5-28065, ThermoFisher).

### Viability Assay

Cells (3×10^3^ cells/well) were seeded in a 96-well plate for 24 h, then treated with 5 μM sorafenib, 20 μM flufenamic acid or 50 μM 5β-Cholanic acid, 25 μM Etomoxir (S8244, Selleck), or 1 μM chloroquine (S6999, Selleck) for either 24 h or 48 h. Cell viability was determined by either CCK-8 assay (C0039, Beyotime, Shanghai, China) or Cell Tilter-Glo Luminescent assay (G7572, Promega, WI, USA).

### Colony Forming Assay

Cells (1×10^4^ cells/well) were seeded in a 6-well plate for 24 h, then treated with indicated drugs for 2-3 weeks. Cells were stained with crystal violet. Representative images were captured using Tanon 2500R Gel Imaging System (Shanghai, China).

### Measurement of mitochondrial membrane potential and ROS

Next, 3×10^5^ cells were incubated with indicated drugs for 24 h. After washing with PBS, cells were stained with 100 nM MitoTracker Red CMXROS (M7512, Thermo Scientific™) or 5 μM MitoSOX Red (M36008, Thermo Scientific™) for 30 min. Stained cells were then immediately captured by confocal microscopy or resuspended in PBS and measured by flow cytometry (BD Cantoli, USA). All FACs data were analyzed by FlowJo software. For total cellular ROS analysis, cells were stained using DCFH-DA (D6883, Sigma), then subjected to fluorescent microscopy.

### Apoptosis Assay

Cells were seeded in a 6-well plate and incubated with drugs for 24 h. After washing with PBS, cells were stained using Alexa Fluor -conjugated Annexin V and propidium iodide (PI) (V13241, Thermo Scientific™) according to the manufacturer`s instructions, followed by a flow cytometric assay (BD Cantoli, USA). Raw data were analyzed by FlowJo software.

### Proteomics

HCC cells (1×10^6^) were lysed in the lysis buffer containing 10% 1-dodecyl-3-methylimidazolium chloride (C_12_Im-Cl) 51 for 24 h and proteins were quantified using the BCA kit (P0012s, Beyotime, Shanghai, China). Proteins were then denatured using dithiothreitol (DTT), subjected to iodoacetamide (IAA) alkylation treatment in the dark for 30 min, and then digested by trypsin at the ratio of 1:30 (enzyme/protein, m/m) overnight at 37 °C. After incubation with 0.1% formic acid (FA), the peptides were analyzed with a Q-Exactive mass spectrometer (Thermo Fisher Scientific, USA).

Peptides were separated in C18 columns with mobile phases including buffer A (98% H_2_O and 2% acetonitrile with 0.1% FA) and B (2% H_2_O and 98% acetonitrile with 0.1% FA). The separation gradient was set as follows: 3-12% B for 65 min and 12-20% B for 65 min, 20-40% B for 5 min, and 40-80% B for 1 min, and maintained for another 15 min. The full MS resolution was 70,000 within the scan range of 300-1800 m/z. dd-MS2 resolution was 17,500 with the fixed first mass of 110 m/z. The loop count was 20, and the top 20 most intense ions were selected with the isolation window of 2.0 m/z. Mass spectrometry raw files were analyzed using the MaxQuant software (1.6.3.3 version) with label-free quantification (LFQ). The mass spectra were aligned with the Uniprot database for humans (42,454 entries). Enzyme specificity was set to trypsin with a maximum of 2 missed cleavages. Fixed modification was set to carbamidomethyl [C] and variable modification was set to Acetyl [N] and oxidation [M]. The identification of peptide-spectrum match (PSM) and protein false discovery rate (FDR) was set at 0.01. For quantification of LFQ intensities and ratios for the proteins, protein group files were extracted from MaxQuant to the Perseus software 52 for analysis.

### Labeling cells with deuterium^3^-palmitic acid

Cells were incubated with a final concentration of 250 μM palmitic acid-16,16,16-d^3^ (615951, Sigma, USA) in serum-free DMEM (C11995500, Gibco, China) for 24 h according to a previously described method 53. Briefly, palmitic acid was dissolved in ethanol and combined with fatty acid-free bovine serum albumin (BSA) at a molar ratio of 10:1 (PA:BSA) in a serum-free medium.

### Lipidomic Analysis

Lipids were extracted from 1×10^6^ cells using the liquid-liquid extraction method 54 containing MeOH, CHCl_3_, and H_2_O. The organic phase was collected and then lyophilized. A reversed-phase BEH C8 column (2.1 mm×100 mm, 1.7 µm, Waters, Milford, MA, USA) was used for the chromatographic separation of lipids. Mobile phases A and B were ACN/H_2_O (60:40, v/v) and IPA/ACN (90:10, v/v), respectively, both containing 10 mM AmAc. The flow rate was 0.3 mL/min. The column temperature was 60 °C. The elution gradient started with 50% B and was kept for 1.5 min, then linearly increased to 85% B at 9 min, and then to 100% B at 9.1 min and held for 1.9 min. The gradient returned to 50% B at 11.1 min and was maintained for 1.9 min to equilibrate column. The temperature of the sample manager was set at 10 °C. Freeze-dried samples were reconstituted in ACN/IPA/H_2_O (65:30:5, v/v/v) containing 5 mM AmAc, and 5 µL was injected into the LC-MS system. The mass spectrometer was operated with a capillary voltage of 3.5 kV in positive mode and 3.0 kV in negative mode. The capillary temperature was set as 300 °C. Sheath gas flow rate and aux gas flow rate were set at 45 and 10 (in arbitrary units). Aux gas heater temperature was 350 °C. The S-lens rf level was 50.0. The resolutions of 120,000 and 30,000 were set for full scan MS and data-dependent MS/MS (ddMS2) in positive mode, and 6×10^5^ and 3×10^5^ in negative mode. The AGC target and maximum IT were 3×10^6^ ions capacity and 100 ms in full scan MS settings while their values were 1×10^5^ ions capacity and 50 ms in ddMS2 settings. The TopN (N, the number of top most abundant ions for fragmentation) was set to 10. The normalized collision energy (NCE) was set. The scan range was set at m/z 300-1250 in positive mode while 160-1600 in negative.

### Plasmids and agents

The full-length cDNA encoding human AKR1C3 was obtained from HepG2 cells and subcloned into a pCDH-CMV-MCS-EF1-Puro vector (CD510B, Addgene, USA). The shRNA of ATG5 were brought from Origne (TR314610, Beijing, China). FUW mCherry-GFP-LC3 plasmid (110060, Addgene, USA) was used for autophagic flux assay. MCherry-LC3 plasmid was kindly gifted by Prof. Zhaochao Xu. The AKR1C3 inhibitor flufenamic acid was bought from Selleck (S4268, USA) which was used 20 μM in the experiments. The AKR1C3 inhibitor 5-β cholanoic acid was bought from Sigma (C7628, USA) and used 50 μM in the experiments.

### Generation of the AKR1C3^-/-^ cell lines

Gene knockout was achieved by Lenti-CRISPR-based method (PMID, 24336571) and single-cell populations were isolated and amplified for the experiments. In brief, sgRNA was designed by GPP Web Portal (https://portals.broadinstitute.org/gpp/public/) and synthesized by GENEWIZ company. sgRNAs were subcloned into a LentiGuide-Puro vector (56963, Addgene) and lentiviruses were packaged and produced in 293T cells. After transfecting into Cas9-expressing cell lines, a single cell was sorted by FACs and selected in 2 μg/mL puromycin. Gene knockout efficiency was verified by protein immunoblotting. The validated sequence of sgRNA for AKR1C3 is shown below: AATGAGCAGAATCTATATGG.

### ECAR and OCR measurements

ECAR and OCR were determined by using the Seahorse Flux Analyzer XF24 and Seahorse Flux Analyzer XF96 according to the manufacturer's instructions. Briefly, 5×10^4^ cells were seeded in either Seahorse 24-wells or 96-wells plates. After 12 h of culture, cells were incubated with indicated treatments for an additional 12 h. The data were normalized by the cell numbers. Glucose, oligomycin (250 nM), 2-DG, FCCP (250 nM), and rotenone / antimycin (100 nM) were added at the indicated time points.

### Xenograft Mouse Model

All animals were bought from Beijing Vital River Laboratory Animal Technology Co., Ltd. Cells (5×10^6^) were mixed with Matrigel (354234, Corning, USA) (1%, v/v) in a total volume of 100 μL, then subcutaneously injected into 10-week-old BALB/c nude mice. Mice were treated with 20 mg/kg sorafenib and/or 20 mg/kg flufenamic acid by intraperitoneal injection for 32 days. Tumor size was measured every 2 days, and tumor volume was calculated using the formula π/6 × length × width^2^.

### Statistical Analysis

All data were represented as mean ± SEM. Statistical analysis was performed using one-way ANOVA or unpaired Student's *t*-test. P value < 0.05 was considered to be statistically significant. All calculations were performed using GraphPad Prism (GraphPad Software, CA, USA) or Excel (Office, USA).

## Supplementary Material

Supplementary figures.
