# Supplementary material for: AKR1C3-dependent lipid droplet formation confers hepatocellular carcinoma cell adaptability to targeted therapy
Source: Theranostics. 2022 Nov 7;12(18):7681–98. doi: 10.7150/thno.74974 (PMC9706585; doi:10.7150/thno.74974)
Supplement: Supplementary file 1 — Supplementary figures. [file thnov12p7681s1.pdf]

Supplementary Materials for

AKR1C3-dependent lipid droplet formation confers hepatocellular carcinoma cells adaptability to targeted therapy

Changqing Wu, Chaoliu Da, Xinyu Li, Mingju Sun, Hongwei Chu, Qiuhui Xuan, Yalei Yin, Chengnan Fang, Fan Yang, Zhonghao Jiang, Qing Lv, Keqing He, Yiying Qu, Baofeng Zhao, Ke Cai, Shuijun Zhang, Ran Sun, Guowang Xu, Lihua Zhang, Siyu Sun, Yang Liu

Correspondence to yliuqq1980@hotmail.com

This PDF file includes:

Supplementary Figures 1-7

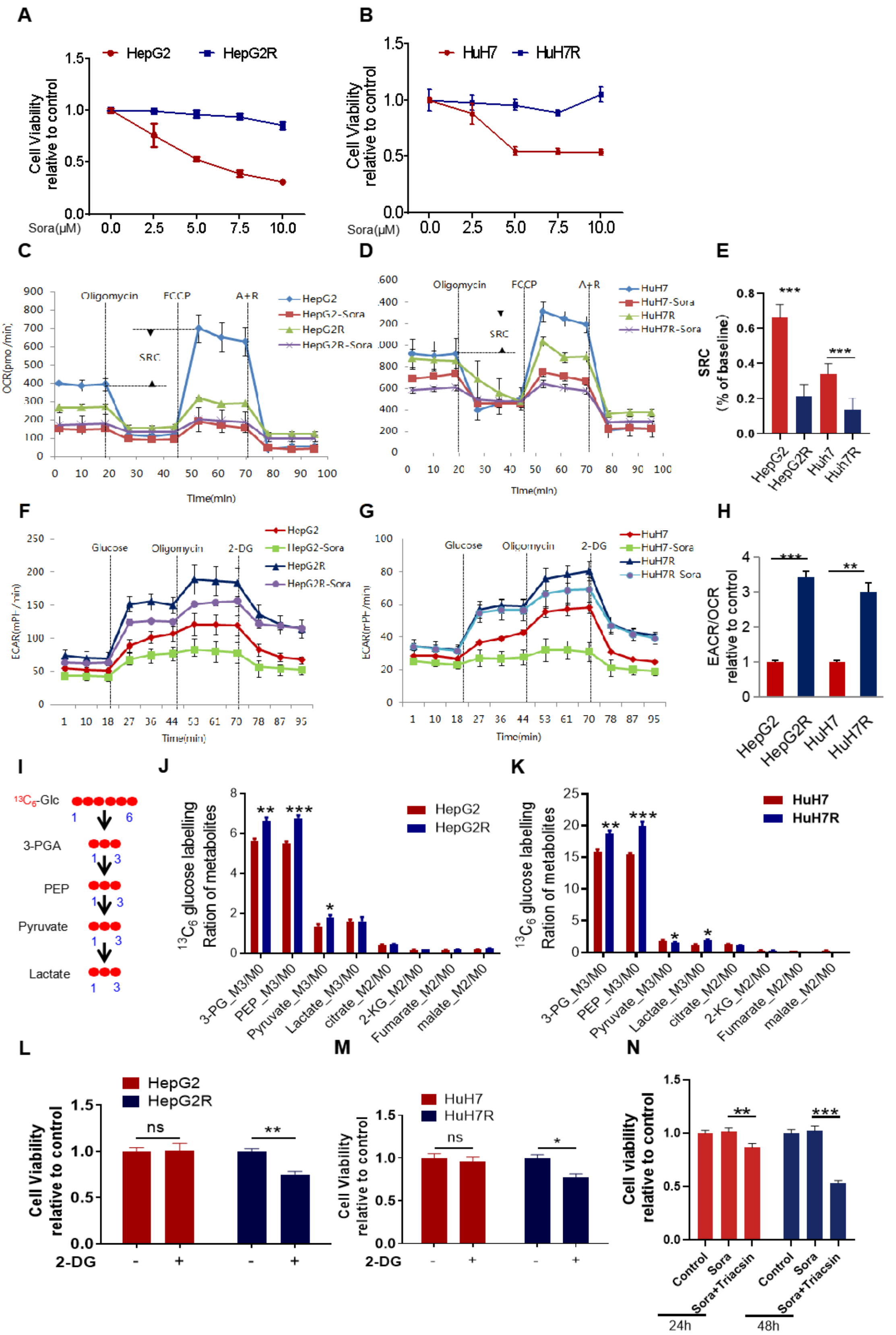

Supplementary Figure 1. Energetic shift from FAO towards glycolysis in resistant HCC cells (A-B) Cell viability was assessed by Cell Titer-Glo assay in the presence of sorafenib for

24 h, respectively. **(C-D)** Oxygen consumption rate (OCR) was measured by Seahorse XF-24 assay in HepG2, HepG2R, HuH7 and HuH7R cells with or without sorafenib treatment, respectively. Oligomycin, FCCP(carbonyl cyanide-4-trifluoromethoxy) and A+R (antimycin and rotenone) were added at the indicated time points. **(E)** Alteration of Spare respiratory capacity (SRC) between maximal uncontrolled OCR (top horizontal dashed line) and the initial basal OCR (bottom horizontal dashed line). **(F-G)** Extracellular Acidification Rate (ECAR) was measured by Seahorse XF-24 assays in these two paired HCC cell lines with or without sorafenib treatment. Glucose, Oligomycin and 2-DG (2-Deoxy-D-glucose) were added at the indicated time points. **(H)** Ratios of ECAR to OCR of the two paired HCC cell lines. **(I)** $^{13}C_6$-labeled glucose fules to glycolytic flux. **(J-K)** Relative quantification of ^{13}C-labeled metabolites to non-^{13}C-labeled metabolites, including 3-PG, PEP, pyruvate, lactate, citrate, 2-KG, fumarate and malate in HepG2, HepG2R, HuH7 and HuH7R cells cultured with $^{13}C_6$-glucose for 24 h. M represents ^{13}C. (**L-M**) Cell viability was measured by cell Titer-Glo assay in HepG2, HepG2R, HuH7 and HuH7R cells in the presence or obscene of 2-DG for 24 h, respectively. (**N**) HepG2R was treatment with sorafenib and Triacsin C for 24 h and 48 h. Cell viability was assessed by Cell Titer-Glo assay. All data are quantified as mean ± SEM. Asterisk indicates significant difference, “n.s” indicates no significance, * $p < 0.05$, ** $p < 0.01$, *** $p < 0.001$.

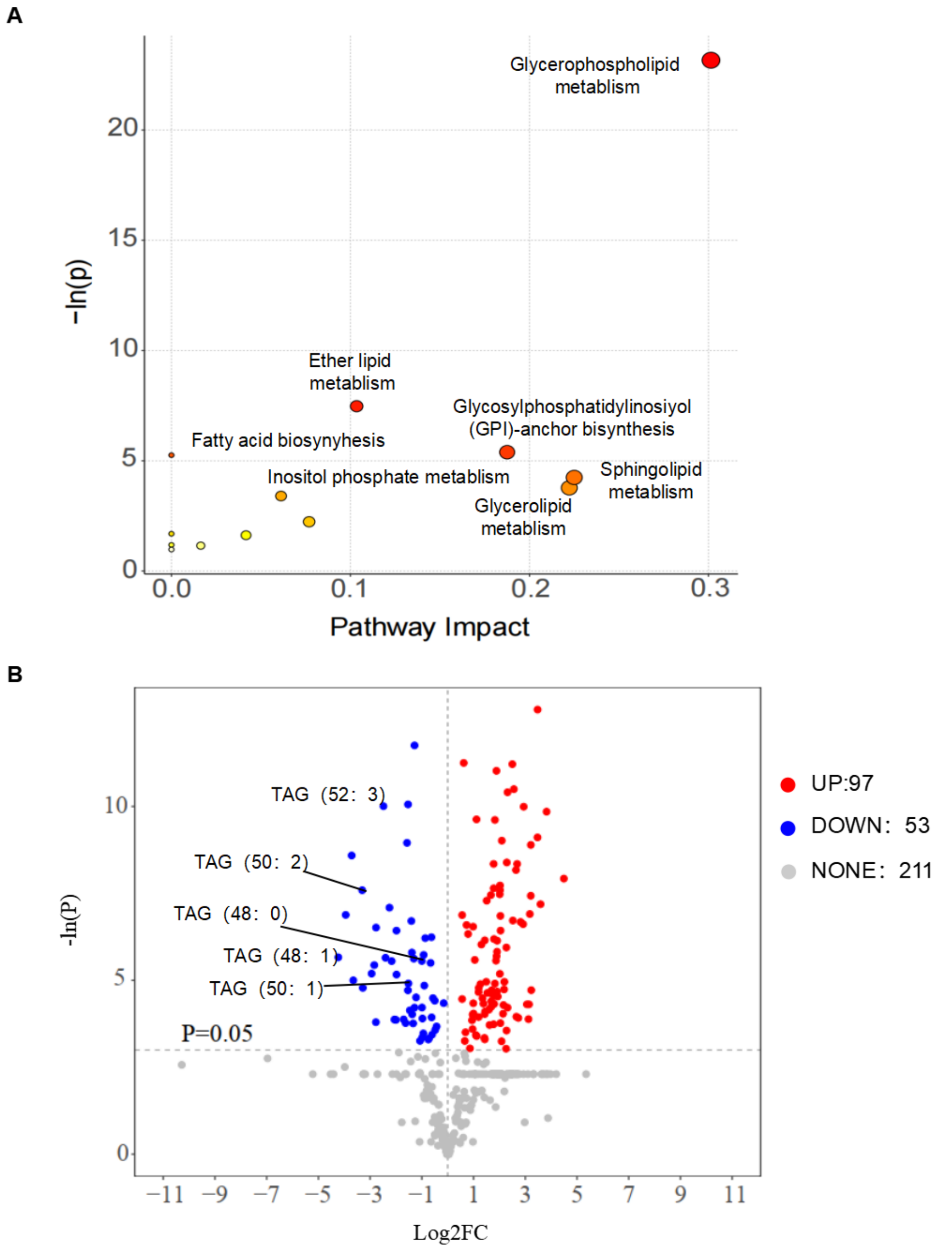

Supplementary Figure 2. Pathway enrichment analysis in AKR1C3$^{-/-}$ cells (A) Altered KEGG metabolic pathways in the Lenti-guide control and AKR1C3$^{-/-}$ HepG2 cell lines were enriched by significantly altered metabolites (FDR < 0.05). **(B)** Volcano plot of TAGs alterations between Lenti-guide control and AKR1C3$^{-/-}$ HepG2 cell lines.

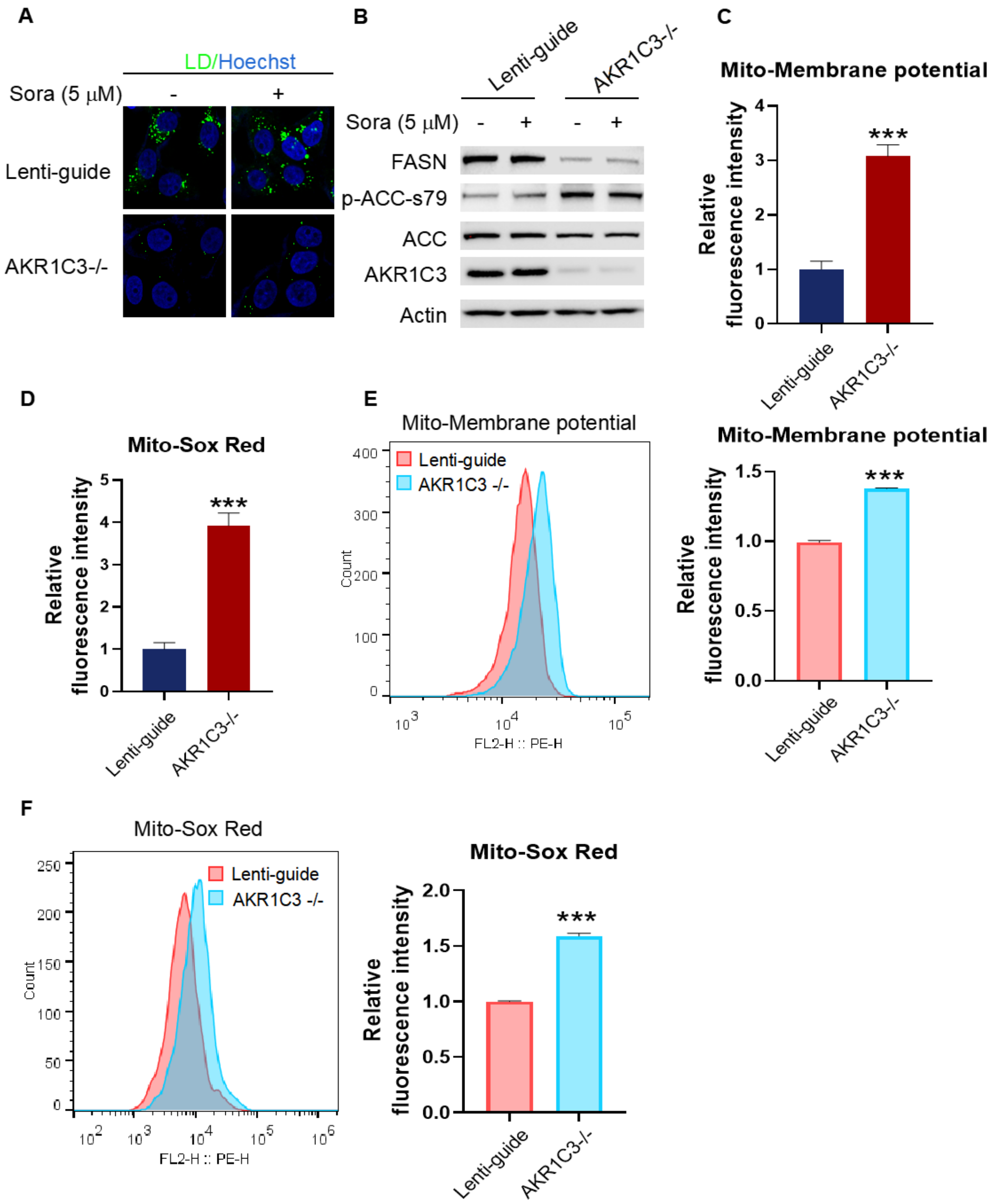

Supplementary Figure 3. Inhibition of AKR1C3 lead to LD breakdown and mitochondrial dysfunction in HuH7R cells (A) HuH7R Lenti-guide and AKR1C3$^{-/-}$ cells were treated with sorafenib, for 24 h, then stained by Bodipy493/503. Cellular LDs contents were visualized by fluorescent microscopy. Nucleus was stained by Hoechst (blue).

(B) The expression of FASN, ACC and p-ACC-s79 and AKR1C3 was assessed by immunblotting. **(C-D)** Statistical analysis of Mito-Membrane potential and Mito-sox Red in HepG2R Lenti-guide and AKR1C3$^{-/-}$ cells. **(E-F)** HuH7R Lenti-guide and AKR1C3$^{-/-}$ cells were

treated sorafenib for 24 h. The cells were stained by CMXROS (red) and Mito-Sox Red, followed by FACs assay.

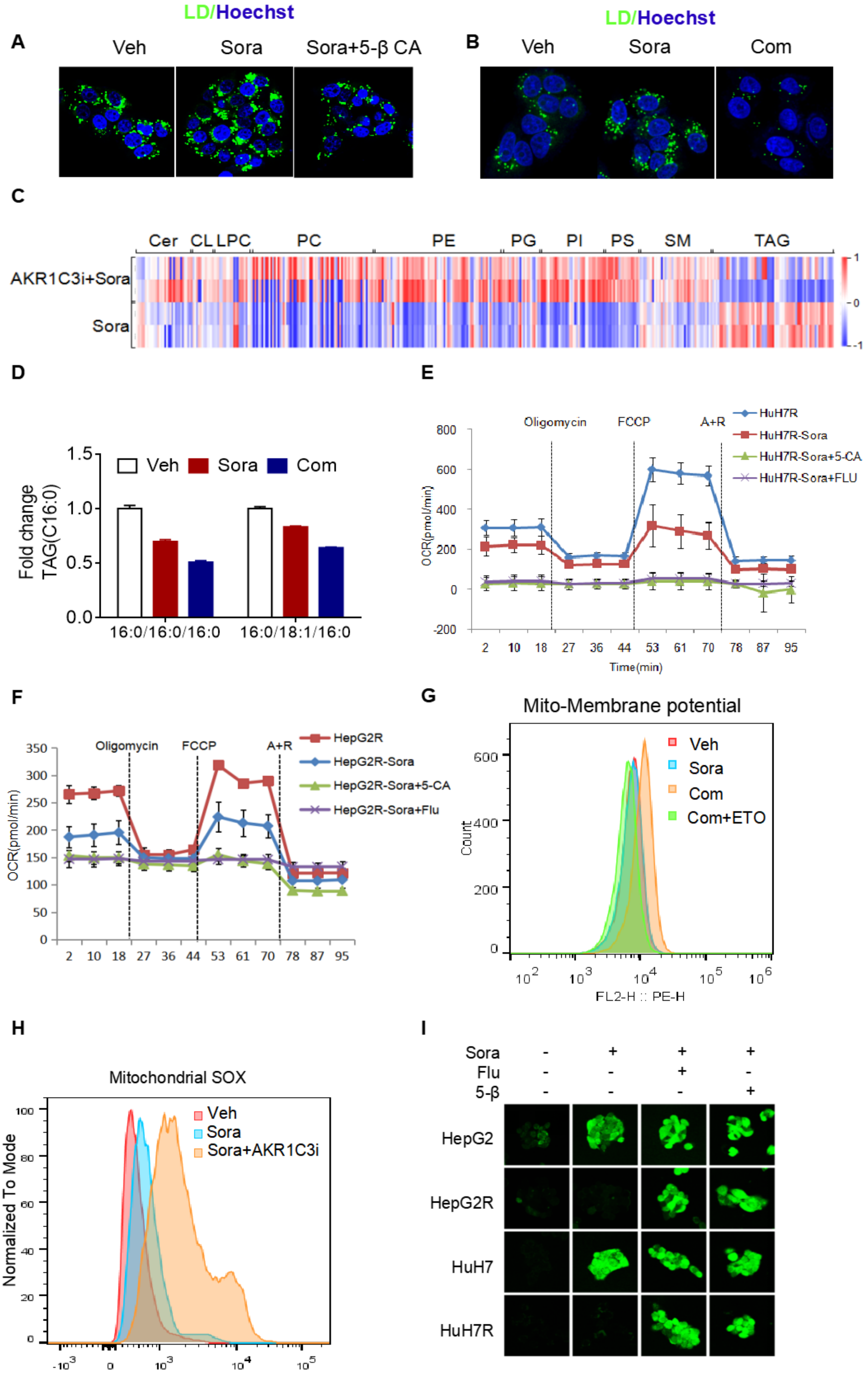

Supplementary Figure 4. LD protects HCC cells from sorafenib-induced mitochondrial lipotoxicity (A) HepG2R cells were treated with vehicle or sorafenib, or combined with

5β-cholanic acid for 24 h, then stained by Bodipy493/503. Cellular LDs contents were visualized by fluorescent microscopy. Nucleus was stained by Hoechst (blue). Com indicates the combination treatment of sorafenib and AKR1C3 inhibitor.**(B)** HuH7R cells were treated with sorafenib, or combined with AKR1C3 inhibitor for 24 h, then stained by Bodipy493/503. Cellular LDs contents were visualized by fluorescent microscopy. Nucleus was stained by Hoechst (blue). **(C)** Heatmap analysis of altered metabolites (FDR < 0.05) in sorefenib resistant HCC cell lines cultured with either sorafenib alone or sorafenib combined with AKR1C3 inhibitor. **(D)** Quantification showing the relative abundance of TAG (C16:0/C16:0/C16:0), TAG (C16:0/C18:1/C16:0). **(E-F)** HepG2, HepG2R, HuH7 and HuH7R cells were treated with or without sorafenib, or with additional AKR1C3 inhibitors, respectively. Oxygen consumption rate was measured by Seahorse XF-24 assay. Oligomycin, FCCP, antimycin and rotenone were added at the indicated time points. **(G)** HuH7R cells were treated with or without sorafenib, or combined with AKR1C3 inhibitor and additional Etomoxir for 24 h. The cells were stained by CMXROS (red), followed by FACs assay. **(H)** HuH7R cells were treated with or without sorafenib, or combined with AKR1C3 inhibitor for 24 h. The cells were stained by Mito-Sox Red, followed by FACs assay. **(I)** HepG2, HepG2R, HuH7 and HuhHR cells were treated with or without sorafenib, or with additional AKR1C3 inhibitor (FLU or 5-β CA) and additional chloroquine. Total cellular ROS were assessed by DCFH-DA staining and subjected to the immunofluorescence microscopy. Representative images were shown.

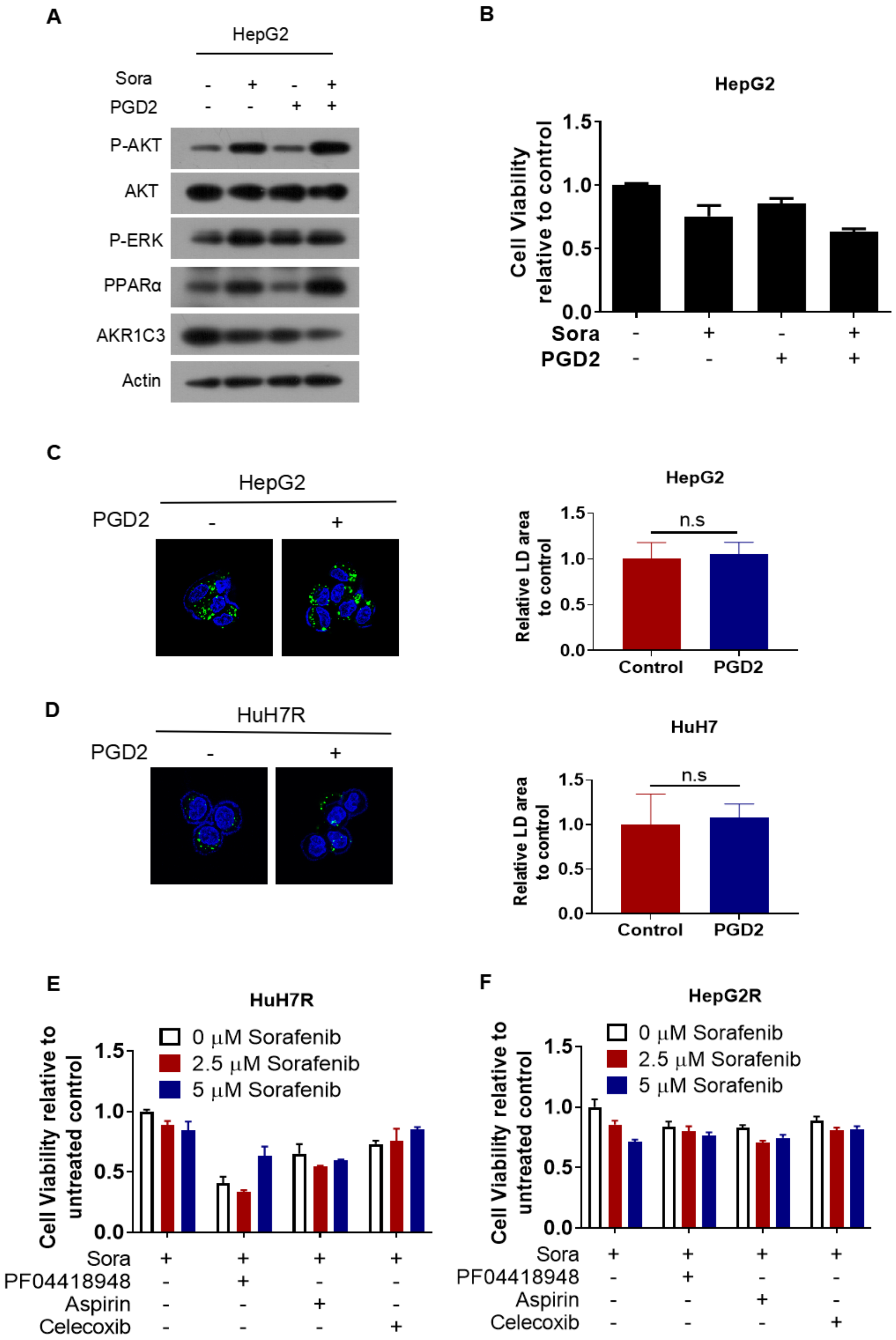

Supplementary Figure 5. AKR1C3 promotes sorafenib resistance and lipid accumulation in a PG-independent manner

(A-B) HepG2 cells were treated with or without sorafenib in the presence or absence of 1 μM PGD2 for 24 h. The extracted protein were analyzed by immunoblotting against the indicated antibodies. Cell viability was assessed by Cell Titer-Glo assay. **(C-D)** HepG2 and HuH7R cells were cultured with or without 1μM PDG2 for 24 h. Bodipy493/503 stained LDs were visualized and analyzed by immunofluorescence microscopy. Nucleus was stained by Hoechst (blue). Histogram plot showed relative LDs area to control in HepG2 and HuH7R. **(E-F)** Cell viability was assessed by Cell Titer-Glo assay in HepG2R and HuH7R cells incubated with sorafenib, or with additional PF04418948, aspirin and celecoxib. All data are quantified as mean ± SEM. Asterisk indicates significant difference. n.s. indicates no significance.

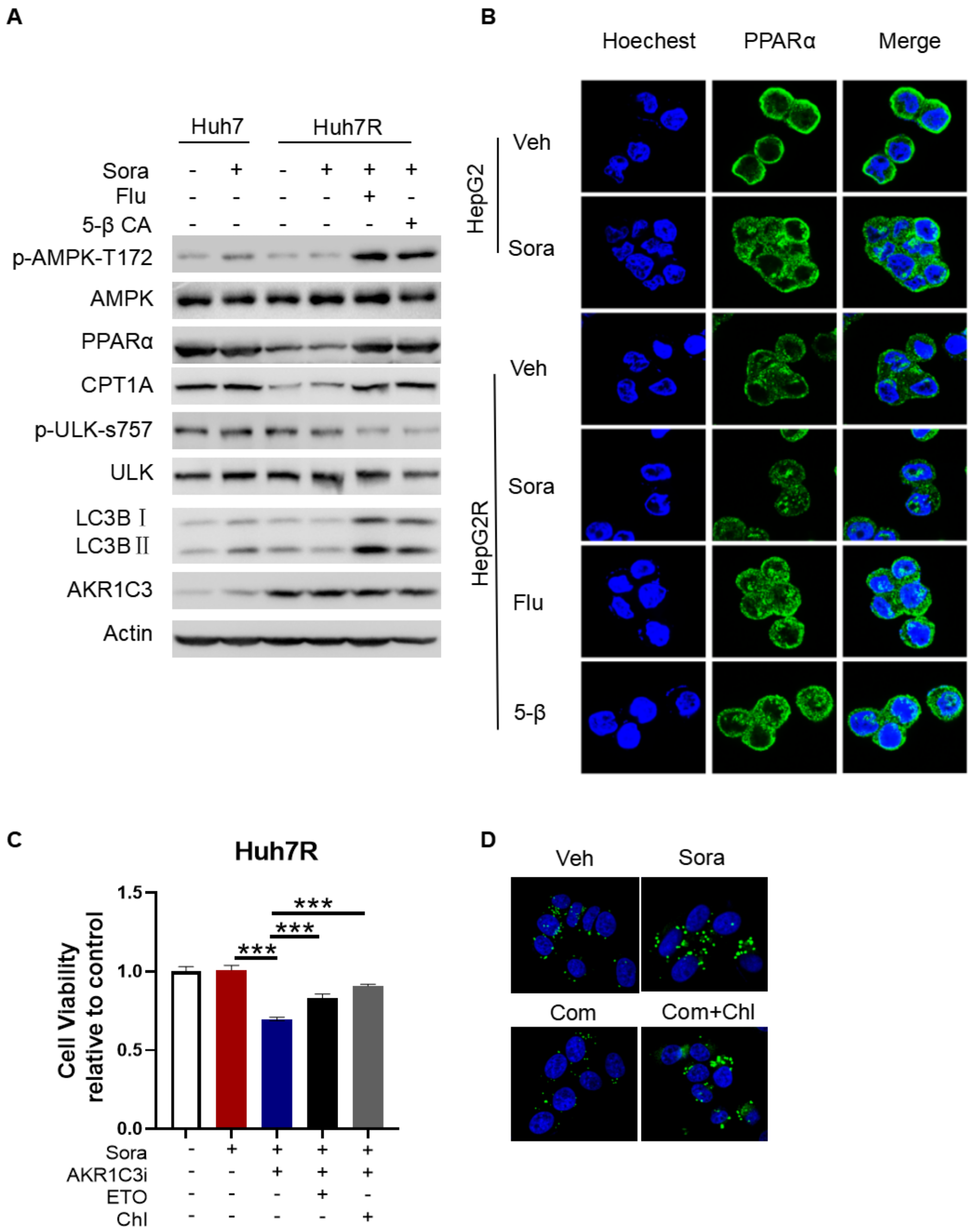

Supplementary Figure 6. AKR1C3 regulates LD homeostasis in HCC cells through lipophagy (A) HuH7and HuH7R cells were treated with Sorafenib and AKR1C3 inhibitors for 24 h. The expression of p-AMPK-T172, AMPK, p-ULK-S757, ULK, CPT1α, PPARα and LC3BI/II was assessed by immunblotting. **(B)** HepG2 and HepG2R cells were treated with or without sorafenib, or with additional Flu and 5-β CA. The nuclear location of PPARα (green) were visualized and analyzed by immunofluorescence microscopy. Nucleus was stained by Hoechst (blue). **(C)** HuH7R cells were treated with sorafenib, or combined with AKR1C3 inhibitor and additional Etomoxir or Chl for 24 h. Cell viability was assessed by Cell Titer-Glo

assay. **(D)** HuH7R cells were treated with sorafenib, or combined with AKR1C3 inhibitor and additional Etomoxir for 24 h, then stained by Bodipy493/503. Cellular LDs contents were visualized by fluorescent microscopy. Nucleus was stained by Hoechst (blue). All data are quantified as mean ± SEM. Asterisk indicates significant difference, *** $p < 0.001$.

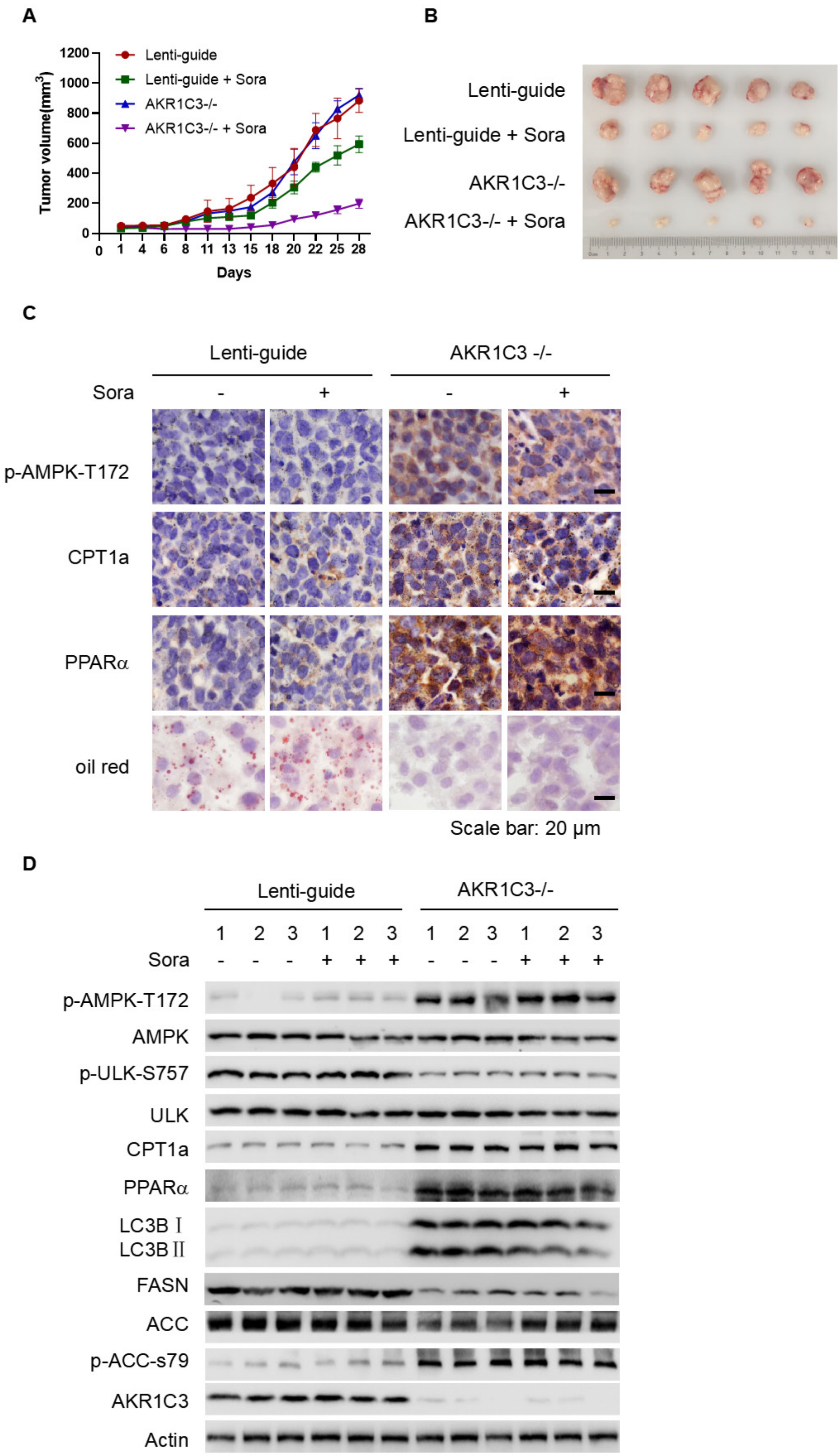
A
B
C
D
Tumor volume(mm3)
Days
Lenti-guide
Lenti-guide + Sora
AKR1C3-/-
AKR1C3-/- + Sora
Lenti-guide
AKR1C3 -/-
Sora
p-AMPK-T172
CPT1a
PPARα
oil red
Scale bar: 20 μm
Lenti-guide
AKR1C3-/-
Sora
p-AMPK-T172
AMPK
p-ULK-S757
ULK
CPT1a
PPARα
LC3B Ⅰ
LC3B Ⅱ
FASN
ACC
p-ACC-s79
AKR1C3
Actin

Supplementary Figure 7. Inhibition of AKR1C3 leads to sorafenib-resistant HCC tumor regression in xenograft mice (A-B) HuH7R Lenti-guide and HuH7R AKR1C3$^{-/-}$ cells were injected into BALB/c nude mice, respectively. Tumor volume was calculated by using caliper measurements ($\pi/6 \times$ length $\times$ width^2). The typical are shown as mean ±s.d. n=5 for each group. Once the subcutaneous tumors reached the volume of 100 mm^3, mice were randomly subjected to vehicle, sorafenib (25 mg/kg), Flufenamic acid (25 mg/kg), or combination of sorafenib and Flufenamic acid given every 2–3 days at the same dose, respectively. Three weeks post-implantation, tumors were isolated from each group. Pictures of isolated tumor are shown in the right. **(C)** Xenograft tumors from each group were sectioned, then subjected to inmmunohistochemistry against the indicated antibodies. Oil red was used to stain lipid droplets in these tumors. Scale bar, 20 μm. The mice were randomly selected from each group. Representative images were shown. **(D)** The expression of p-AMPK-T172, AMPK, p-ULK-S757, ULK, CPT1a, PPARα, LC3B I/II, FASN, ACC, p-ACC-s79 and AKR1C3 were assessed by immunblotting.
